# Analysis of Unmanned Aerial Vehicle Compromising Emissions in Terms of the Possibility of Reconstructing the Transmitted Image

**DOI:** 10.3390/s26196142

**Published:** 2026-09-28

**Authors:** Rafał Przesmycki, Marek Bugaj, Bartosz Dudziński, Michał Kryk

**Affiliations:** Department of Electronics, Military University of Technology, 00-908 Warsaw, Poland; marek.bugaj@wat.edu.pl (M.B.); bartosz.dudzinski@wat.edu.pl (B.D.); michal.kryk@wat.edu.pl (M.K.)

**Keywords:** UAV, compromising emissions, Autel, DJI, electromagnetic emissions

## Abstract

This article presents the results of research on compromising emanations generated by unmanned aerial vehicles (UAVs) in the context of potential electromagnetic eavesdropping and the reconstruction of transmitted video data. Two commercial UAV systems were analyzed: the Autel EVO II Pro and the DJI Mavic 3. The aim of the research was to identify the electromagnetic characteristics of unintentional emissions recorded during the transmission of selected imagery and to assess their suitability for reconstructing the information processed by the UAV system. The measurements were conducted in an anechoic chamber using specialized measurement equipment from INTRIPLE. Two variants of the system configuration were considered: the first involved standard image transmission between the UAV platform and a controller equipped with a display, while the second comprised an extended configuration where an additional TV receiver was connected to the controller via an HDMI interface. The recorded signals were analyzed to extract features, enabling the identification of correlations between the electromagnetic emissions and the transmitted video content. The results indicate the presence of characteristic components of compromising emanations, with properties depending on both the UAV platform used and the configuration of the image transmission path. The analysis confirms the need for further research into UAV susceptibility to electromagnetic eavesdropping and the potential for signal reconstruction from unintentional emissions.

## 1. Introduction

The dynamic development of unmanned aerial vehicle (UAV) technology has made these systems an integral part of contemporary civilian, industrial, and military applications. Initially used primarily for military operations and reconnaissance, UAVs are now widely employed in areas such as critical infrastructure monitoring, surveying, precision agriculture, search and rescue operations, technical inspections, public safety, photography, and audiovisual transmission. Modern unmanned platforms are equipped with high-resolution imaging systems, advanced flight stabilization mechanisms, and efficient data transmission systems that enable real-time image transmission to ground operators.

However, the increasing functionality of UAV systems is accompanied by a growing number of threats related to the security of the information being processed and transmitted. In particular, real-time image transmission between the aerial platform and the operator station is critical for both operational security and data confidentiality. The interception of visual information transmitted by UAVs may reveal reconnaissance data, object locations, mission progress, or environmental parameters recorded during mission execution. Consequently, the electromagnetic security of unmanned systems has become a crucial issue not only for military applications but also for civilian infrastructure and commercial systems.

Research on UAV security primarily focuses on resilience against radio interference, cyberattacks, communication channel hijacking, GNSS signal spoofing, and the vulnerability assessment of wireless communication protocols. Comparatively little attention has been devoted to threats arising from unintentional electromagnetic emissions generated by systems responsible for processing and transmitting visual information. Nevertheless, every electronic device, especially those incorporating digital signal processing components, emits a certain amount of electromagnetic radiation due to the operation of logic circuits, data buses, communication interfaces, and display subsystems [1,2,3].

The phenomenon of unintentional electromagnetic emissions that may lead to information disclosure is referred to as compromising emanations. This issue forms the foundation of the field of electromagnetic security, often referred to as TEMPEST or Emission Security (EMSEC), which encompasses the analysis of the potential for obtaining information from the unintentional emissions of electronic devices. Research in this area has demonstrated the feasibility of reproducing information displayed on computer monitors, reconstructing data transmitted through digital interfaces, and identifying content processed by ICT devices solely on the basis of recorded electromagnetic emissions. Graphics processing and image transmission systems are particularly susceptible, as their time-frequency characteristics often reflect the structure of the visual data being processed.

Countermeasures against compromising electromagnetic emanations are already well established within the EMSEC/TEMPEST domain. However, their effectiveness remains dependent on the specific system architecture and implementation, particularly when additional high-speed interfaces and external display devices are introduced.

In the case of UAV systems, the issue of compromising emanations may be particularly significant due to the nature of the tasks performed and the architecture of the image transmission system. A typical unmanned system consists of an aerial platform equipped with a camera and image encoding modules, a wireless data transmission channel, and an operator control station with a display that enables real-time image viewing. Each of these components may constitute a potential source of unintentional electromagnetic emissions which, when combined with appropriate measurement and analytical techniques, may enable the partial or complete reconstruction of the transmitted visual information. Furthermore, the use of external image interfaces, such as HDMI, may alter the emission characteristics of the system and increase the risk of unauthorized acquisition of sensitive information.

Despite the growing importance of unmanned systems and the rapid development of electromagnetic reconnaissance techniques, the vulnerability of UAV systems to electromagnetic eavesdropping remains relatively underexplored. In particular, only limited research has investigated the practical feasibility of identifying and reconstructing images transmitted by commercial UAV platforms through the analysis of compromising emanations. Therefore, further experimental studies are required to determine the electromagnetic characteristics of such systems and to assess the potential risk of information leakage.

The scientific problem addressed in this study is not the general feasibility of recovering visual information from compromising electromagnetic emanations, which has already been demonstrated for other electronic systems, but the configuration-dependent susceptibility of commercial UAV image-presentation systems. In particular, the study investigates how the architecture of the reception, decoding, rendering, and display chain influences the amount of recoverable spatial information.

The following sentences concerning the applied problem and the characterization of the experiment as a controlled laboratory proof-of-concept are retained. The manuscript already explicitly states that the work is a baseline susceptibility assessment rather than an operational interception-system demonstration.

The aim of this article is to analyze compromising emanations generated by selected commercial UAV systems in the context of image reconstruction. The study was conducted on two widely used unmanned platforms: the EVO II Pro by Autel Robotics and the Mavic 3 by DJI. The analysis focused on unintentional electromagnetic emissions recorded during the transmission of selected images under various transmission system configurations. Both a standard configuration, involving communication between the UAV platform and an operator control station equipped with a display, and an extended configuration, in which the controller was connected to an external television receiver via an HDMI interface, were investigated. The scope of the study included the identification of characteristic components of electromagnetic emissions, an analysis of the relationship between the properties of the recorded signals and the transmitted visual content, and an assessment of the potential for reconstructing image information. The results presented in this paper may provide a foundation for further research on the electromagnetic security of UAV systems and the development of methods aimed at reducing the risk of unauthorized information acquisition through electromagnetic eavesdropping techniques.

Accordingly, the contribution of this work is primarily an experimental assessment of configuration-dependent electromagnetic information leakage in real commercial UAV systems rather than a generic demonstration of the feasibility of electromagnetic image reconstruction.

## 2. Compromising Emanations and the Possibility of Information Reconstruction

Modern electronic systems responsible for data processing, transmission, and presentation inherently generate electromagnetic emissions as a by-product of the operation of their electronic components. These emissions may be classified as either intentional, associated with the communication functions of the device, or unintentional, arising from electrical activity within digital circuits, signal lines, data buses, and communication interfaces. Under certain circumstances, such unintentional emissions may exhibit a correlation with the information being processed. Consequently, they may constitute a potential source of information leakage, enabling unauthorized acquisition of sensitive data without requiring physical access to the system.

### 2.1. The Nature of Compromising Emanations

The phenomenon of electromagnetic emissions that enable the disclosure of information processed by a device is referred to as compromising emanations. This term refers to any signals unintentionally emitted by electronic devices, the analysis of which may lead to the reconstruction of processed data, the identification of system activity, or the determination of the characteristics of performed operations. In practice, compromising emanations constitute a specific category of information security threats, as they enable the application of passive technical surveillance techniques without interfering with the structure or operation of the investigated system [4,5].

This issue is addressed within the field of electromagnetic security (EMSEC), often associated with the concept of TEMPEST, which encompasses methods for protecting information against unauthorized acquisition through electromagnetic emissions. Research on compromising emanations dates back to the Cold War, when it was demonstrated that information processed by cryptographic devices, computer terminals, and telecommunications systems could be reconstructed solely on the basis of recorded electromagnetic emissions. Since then, this field has expanded significantly to include the analysis of emissions generated by personal computers, displays, printers, mobile devices, data transmission interfaces, and wireless communication systems.

The primary source of these emissions is digital circuitry undergoing rapid logical state transitions. Variations in voltage and current levels within conductors and printed circuit boards generate electromagnetic fields whose time-frequency characteristics may reflect the nature of the data being processed. In particular, high-frequency signals found in graphics processing and multimedia transmission systems often exhibit a strong correlation with the information content of the original signal [4,5].

### 2.2. Mechanisms of Electromagnetic Emission Generation

The generation of electromagnetic emissions in digital devices results from fundamental physical phenomena associated with the flow of electric current and the switching of logical states within electronic circuits. Each transition of a transistor between logical states produces short-duration current pulses that generate electromagnetic components propagating through both radiated and conducted paths.

In practice, emissions may originate from various components of an electronic system, including data buses, clock and synchronization circuits, digital signal processors, memory modules, transmission cables, high-speed digital interfaces, and image display subsystems.

Image processing systems constitute a particularly significant source of compromising emanations, as the structure of visual data directly influences the behavior of electrical signals. Image brightness, contrast, and the presence of edges, text, or geometric features may cause variations in the characteristics of electromagnetic emissions. As a result, correlations may exist between the recorded electromagnetic signals and the properties of the processed visual content.

The influence of transmission interfaces on the level and nature of electromagnetic emissions is another important aspect. Modern image transmission standards, such as HDMI, DisplayPort, and LVDS, employ high-speed differential signaling with substantial bandwidth requirements. Despite the implementation of mechanisms designed to reduce electromagnetic emissions, these interfaces may still constitute sources of compromising emanations. Under certain conditions, signals emitted by transmission cables can be intercepted and subsequently analyzed to reconstruct the transmitted information [4,5,6].

### 2.3. Reconstruction of Information from Electromagnetic Emissions

One of the most important aspects of research on compromising emanations is the ability to reconstruct information processed by a device on the basis of recorded electromagnetic signals. This process involves identifying emission components that contain information about the structure of the source data and subsequently applying appropriate signal analysis techniques to reconstruct that information.

In the case of image display systems, reconstruction may involve reproducing screen content through the analysis of signals synchronized with the display refresh rate and frame structure. The literature has demonstrated the possibility of obtaining readable images from CRT, LCD, and LED monitors solely on the basis of electromagnetic emissions recorded at distances exceeding several tens of meters. In many cases, it was possible to identify text, symbols, and graphical details displayed on the screen.

The information reconstruction process typically involves several stages:

1. Electromagnetic signal acquisition—using broadband antennas and measurement receivers.
2. Frequency-domain filtering and selection—identifying frequency bands containing components correlated with useful information.
3. Time synchronization—determining the display refresh rate or system clock frequency.
4. Signal processing—extracting relevant features and improving the signal-to-noise ratio.
5. Image reconstruction—recovering visual information using analog or digital techniques.

The effectiveness of the reconstruction process depends on numerous factors, including the emission level, signal bandwidth, modulation scheme, measurement distance, environmental noise level, and the degree of synchronization of the source signal. In practice, complete information reconstruction is not always possible. However, even partial image recovery or the identification of characteristic visual elements may result in a significant compromise of information security [6,7,8,9,10].

### 2.4. Compromising Emanations in UAV Image Transmission Systems

UAV systems constitute a particularly interesting subject of research in the field of compromising emanations due to their complex architecture for processing and transmitting visual data. A typical unmanned system performs a multi-stage process that includes image acquisition by an onboard camera, video signal encoding, radio transmission to the operator station, image decoding, and display on the controller screen or an external display device.

Each of these stages may constitute a potential source of unintentional electromagnetic emissions. Emissions generated by the operator control station and image transmission interfaces are of particular interest, as they process data after video signal decoding, which may increase the correlation between electromagnetic emissions and the displayed visual content. When additional display devices, such as monitors or televisions connected via an HDMI interface, are used, the number of potential sources of compromising emanations increases further.

It should also be emphasized that commercial UAV systems are primarily designed with transmission efficiency, image quality, and user experience in mind, whereas electromagnetic security is often not considered a primary design objective. Consequently, the actual susceptibility of such systems to electromagnetic eavesdropping may remain insufficiently understood, thereby justifying the need for further experimental research in this area.

### 2.5. Current State of Research on Compromising Emanations

To date, research on compromising emanations has focused primarily on conventional ICT systems, computer monitors, mobile devices, and wired transmission interfaces. The literature has demonstrated the feasibility of image reconstruction for various display technologies, as well as the possibility of extracting information from signal cables and peripheral devices.

Significantly less attention has been devoted to the analysis of electromagnetic emissions generated by unmanned aerial systems, particularly in the context of real-time image transmission. The limited number of available studies primarily address radio channel security and resistance to interference, while largely overlooking the issue of unintentional electromagnetic emissions and the potential reconstruction of visual information. As a result, a significant research gap exists regarding the assessment of the susceptibility of modern UAV platforms to electromagnetic information leakage.

Addressing this gap requires controlled experimental studies aimed at identifying the characteristic features of electromagnetic emissions generated by real UAV platforms and assessing the relationship between the recorded signals and the transmitted image content. This challenge constitutes the primary motivation for the research presented in this article.

## 3. Characteristics of Selected UAVs

To investigate compromising emanations and the possibility of reconstructing transmitted images, two commercial unmanned aerial vehicle (UAV) platforms representing an advanced class of systems used in professional and surveillance applications were selected. The Autel EVO II Pro [11] and DJI Mavic 3 [12] platforms were analyzed. Both systems are equipped with real-time image transmission capabilities and digital communication links that enable simultaneous flight control, telemetry exchange, and video transmission.

The selection of these platforms was based on their widespread use, high level of technological sophistication, and the implementation of modern radio communication systems operating primarily in the 2.4 GHz and 5.8 GHz frequency bands. From the perspective of electromagnetic security, these systems constitute an interesting subject of research because they incorporate numerous electronic subsystems responsible for image processing, transmission, and display, which may generate unintentional electromagnetic emissions correlated with the transmitted visual content. The following subsections present the technical characteristics of the investigated platforms, with particular emphasis on the data transmission architecture, operating frequencies, and potential sources of electromagnetic emissions relevant to the conducted research.

### 3.1. Characteristics of the Autel EVO II Pro Platform

The Autel EVO II Pro belongs to a class of multirotor unmanned aerial vehicle (UAV) platform designed for professional and semi-professional applications. The platform is widely used for infrastructure monitoring, photogrammetric surveying, search and rescue operations, and terrain reconnaissance, among other applications. The EVO II Pro is equipped with a high-resolution imaging system, including a 6K camera based on a 1-inch CMOS sensor [13], and an image transmission system that enables real-time video streaming to the operator control station. It should be emphasized that the 6K specification refers to the maximum recording capability of the onboard camera and does not represent the resolution of the live video stream analyzed in the present experiments. During the measurements, the image transmitted to the operator controller was configured at 1080i with a frame rate of 50 fps. This setting was kept unchanged throughout the corresponding measurement series. The Autel EVO II Pro platform and its controller are shown in Figure 1.

From the perspective of electromagnetic security and the analysis of compromising emanations, the data transmission architecture between the aerial platform and the ground station is of particular importance. The EVO II Pro employs a bidirectional communication link that simultaneously supports the transmission of control commands, telemetry data, and a high-resolution video stream. Depending on the hardware version, operating region, and software configuration, the system may operate in the 2.4 GHz and 5.8 GHz frequency bands, using dynamic channel selection and adaptive transmission parameter control mechanisms to optimize communication reliability and quality.

The transmission system employed by the Autel platform utilizes a multichannel, high-bandwidth digital communication architecture, enabling the transmission of HD (1080i) video depending on the camera configuration and operating mode. From the perspective of electromagnetic eavesdropping, this implies the presence of multiple potential sources of electromagnetic emissions. These include both radio modules operating in the microwave frequency range and internal video-processing components, such as image encoding units, digital signal processors, and display interfaces integrated into the controller.

A significant feature of the system is the use of adaptive transmission mechanisms, whereby radio parameters are automatically adjusted according to channel quality, interference levels, and the distance between the UAV and the controller. In practice, this enables the dynamic modification of operating frequencies and channel bandwidth. From the perspective of electromagnetic emission analysis, such mechanisms may influence the temporal variability of the spectral characteristics of signals recorded in the vicinity of the system.

In a typical configuration, control and image transmission are implemented within a common radio communication subsystem, while individual data streams remain logically separated at the communication protocol level. The control channel is responsible for transmitting flight commands, operational parameters, and telemetry information, whereas the image transmission channel provides real-time delivery of the video stream. Telemetry data, including altitude, speed, battery status, and geographic position, may be transmitted simultaneously with video data, thereby increasing the overall electromagnetic activity of the system.

In the case of the EVO II Pro platform, the operator controller constitutes a particularly interesting element from the perspective of compromising emanations research. This device performs radio signal reception, video stream decoding, image rendering, and image display functions. Each of these processes involves high-speed digital operations that may generate electromagnetic emissions potentially correlated with the visual content currently displayed on the screen. When an external video output, such as an HDMI interface, is utilized, additional electromagnetic components associated with high-bandwidth digital transmission may also be generated.

According to the technical specifications, the primary intentional emissions associated with radio communication are concentrated in the 2.4 GHz (2400–2483.5 MHz) and 5.8 GHz (5725–5850 MHz) frequency bands [13]. However, compromising emanations may also occur outside these ranges, including harmonics, intermodulation products, and secondary signals generated by digital image-processing circuits. In practice, this necessitates broadband electromagnetic monitoring covering both frequency bands directly associated with radio transmission and those resulting from the operation of internal electronic subsystems.

### 3.2. Characteristics of the DJI Mavic 3 Platform

The DJI Mavic 3 is a technologically advanced commercial UAV platform widely used in professional imaging, inspection, mapping, and monitoring applications. The platform is equipped with a high-performance optical system, including a primary camera featuring a 20 MP 4/3 CMOS sensor [12], and a digital image transmission system that enables the delivery of high-resolution video to the ground operator while maintaining a highly stable communication link. The DJI Mavic 3 platform and its controller are shown in Figure 2.

A key element of the system architecture is the DJI O3+ (OcuSync 3+) transmission technology [14], which provides bidirectional data exchange between the UAV and the operator control station. This system integrates flight control, telemetry, and video transmission within a unified communication environment, providing high throughput and improved link stability under varying radio conditions. The platform primarily operates in the 2.4 GHz and 5.8 GHz frequency bands, with available channel settings depending on the regulatory region and hardware configuration.

The DJI Mavic 3 system employs an automatic channel selection mechanism that dynamically selects the frequency offering the most favorable propagation conditions and the lowest level of interference. This solution enhances transmission reliability; however, it may also result in temporal variations in the electromagnetic characteristics of the system under investigation.

From the perspective of electromagnetic emission analysis, the high bitrate of image transmission is of particular importance. The video stream transmitted between the UAV and the controller contains a large volume of data, the decoding of which requires intensive digital processing at the receiving end. These operations are performed by dedicated digital signal processing (DSP) units and graphics processors responsible for image rendering on the controller display.

Within the operator controller, the received radio signal is converted into a visual image presented to the user. This process includes digital stream decoding, frame synchronization, color reconstruction, and image rendering on the display. Each of these operations may generate unintentional electromagnetic emissions correlated with the visual content of the transmitted image. In the extended experimental configuration, an additional source of electromagnetic emissions was the HDMI interface used to transfer the image signal to an external television receiver. This interface utilizes high-speed TMDS (Transition-Minimized Differential Signaling) technology, operating at frequencies ranging from hundreds of megahertz to several gigahertz, depending on the image resolution and transmission parameters. In practice, this means that additional spectral components associated with digital image transmission may be generated, potentially increasing the susceptibility of the system to electromagnetic eavesdropping.

As in the case of the Autel EVO II Pro platform, the primary intentional emissions associated with radio communication in the DJI Mavic 3 system are concentrated in the 2.4 GHz and 5.8 GHz frequency bands. However, compromising emanations may occur across a much broader frequency spectrum. Their sources include digital circuits responsible for video decoding, graphics processing, and display synchronization.

## 4. Research Methodology

The experimental methodology used to investigate compromising electromagnetic emissions generated during UAV image transmission is described in the following sections. Particular attention was devoted to ensuring repeatable measurement conditions and to comparing the investigated system configurations. The research focused on the analysis of unintentional electromagnetic emissions recorded in the UAV operator controller, which constitutes the final element of the image transmission chain. To ensure the repeatability of results, a controlled experimental environment was developed enabling the measurement of radiated emissions across a wide frequency range.

### 4.1. Object of the Study

To investigate compromising emanations and the possibility of reconstructing transmitted images, two commercial unmanned aerial vehicle (UAV) platforms representing the advanced segment of professional and semi-professional UAV systems were selected. The choice of research objects was based on their high level of technological sophistication, widespread use in operational applications, and the implementation of modern real-time image transmission systems.

The Autel EVO II Pro (Autel Robotics Co., Ltd., Shenzhen, China) and DJI Mavic 3 (SZ DJI Technology Co., Ltd., Shenzhen, China) platforms, described in detail in Section 3, were investigated. Both platforms belong to the class of multirotor UAVs equipped with high-resolution imaging systems and digital communication links. These systems enable real-time image transmission between the aerial platform and the operator controller while simultaneously supporting flight control, telemetry exchange, and video communication functions. 

Regarding electromagnetic security, these platforms constitute particularly interesting research objects due to the complexity of their information-processing architecture. A typical UAV system incorporates numerous electronic subsystems capable of generating unintentional electromagnetic emissions, including radio communication modules, digital signal processors, image encoding and decoding units, data transmission interfaces, and image display systems integrated into the operator controller. Consequently, electromagnetic emissions potentially correlated with the processed visual content may be generated.

Another important criterion for selecting the analyzed platforms was the use of advanced radio transmission systems operating in the microwave frequency range and employing adaptive frequency selection and dynamic channel management mechanisms. In both systems, image transmission and flight control are implemented through digital communication links operating primarily in the 2.4 GHz and 5.8 GHz frequency bands. These mechanisms may influence both the characteristics of intentional radio emissions and the secondary emissions generated by electronic circuits responsible for signal processing.

It should be emphasized that modern commercial UAV systems are primarily designed to ensure high image quality, reliable communication, and ease of operation, whereas protection against electromagnetic eavesdropping and the reduction of compromising emanations are rarely considered primary design objectives. Consequently, the actual susceptibility of such platforms to unauthorized information acquisition through electromagnetic reconnaissance techniques remains insufficiently explored.

During the study, electromagnetic emissions generated by the operator controller were analyzed. The controller constitutes the final stage of the image processing and presentation chain and was therefore assumed to be the system component most likely to generate emissions highly correlated with the transmitted visual content. This assumption results from the fact that the controller is responsible for video signal decoding, image rendering, and visual presentation.

During the experiments, the UAVs remained stationary and were not operated in flight. Each platform was positioned against the wall of the anechoic chamber at a sufficient distance from the measurement setup. The onboard camera was directed toward a prepared test image, which was subsequently transmitted via the radio communication link to the operator controller for display and analysis.

### 4.2. Measurement Setup

The tests were conducted in laboratory conditions, in an anechoic chamber, which limited the impact of external electromagnetic interference and minimized radio wave reflections from environmental elements. The use of a controlled environment allowed for increased accuracy in identifying emissions generated by the UAV system under test and improved the signal-to-noise ratio. A block diagram of the laboratory stand for detecting discrete emissions generated by an unmanned aerial vehicle during image transmission is shown in Figure 3, while Figure 4 shows a view of the laboratory stand for detecting discrete emissions generated by an unmanned aerial vehicle during testing.

The UAV was positioned against the opposite wall of the anechoic chamber, while the operator’s controller was located in the measurement area, directly adjacent to the receiving antenna. This configuration allowed us to limit the impact of emissions directly originating from the aerial platform and focusing the measurement on signals generated by the receiving device.

Electromagnetic emissions were recorded using a specialized INTRIPLE IZ27B measurement receiver operating in conjunction with a set of measurement antennas supplied by the same manufacturer. The receiver enabled broadband analysis of electromagnetic signals within the frequency range from 1 MHz to 12 GHz, allowing the observation of both low-frequency components generated by digital circuits and emissions associated with high-speed data transmission interfaces and radio communication modules.

Measurements were performed exclusively for radiated emissions recorded at a distance of 1 m from the UAV controller, in accordance with the established experimental procedure. The controller was selected as the primary measurement object because it performs the functions of receiving, decoding, and displaying images transmitted from the UAV platform.

To improve the completeness of the electromagnetic signal analysis, measurements were conducted using both vertical and horizontal antenna polarizations. Considering both polarizations made it possible to identify direction-dependent components of electromagnetic emissions and increased the likelihood of detecting signals containing information correlated with the transmitted image.

### 4.3. Experimental Scenarios

The experiments were performed under two image transmission system configurations that differed in the architecture of the visual signal processing chain. The first configuration represented the standard operating mode of the UAV system, in which the image was transmitted directly from the aerial platform to the operator controller equipped with an integrated display. The live-view resolution and frame-rate settings were maintained unchanged for all test images within a given platform in order to avoid introducing additional variability into the electromagnetic-emission characteristics.

Figure 5 presents a schematic diagram of the laboratory stand used for the investigation of electromagnetic emissions in scenario I, corresponding to the standard image transmission configuration. A photograph of the laboratory stand used during the measurements is shown in Figure 6.

The data flow chain in this configuration consisted of the following elements:

test image → UAV camera → radio transmission link → UAV controller → controller display.

In this configuration, the controller served as the final receiving device and displayed the image transmitted by the onboard camera without the use of any additional external equipment. The purpose of the measurements was to determine the characteristics of the electromagnetic emissions generated during standard system operation.

The second experimental scenario involved extending the basic image transmission chain by incorporating an external television receiver connected to the controller via an HDMI interface. Figure 7 presents a schematic diagram of the laboratory stand used for investigating electromagnetic emissions generated during UAV image transmission in scenario II—an extended image transmission path incorporating an HDMI interface. Figure 8 shows the experimental setup used during measurements for this scenario.

The data flow chain in this configuration was as follows:

test image → UAV camera → radio transmission link → UAV controller → HDMI interface → television receiver

The inclusion of an additional system component was intended to assess the impact of a digital image transmission interface on the characteristics of compromising emanations. It was hypothesized that the presence of a high-speed HDMI interface could result in the generation of additional electromagnetic components exhibiting a stronger correlation with the transmitted visual content.

The comparison of both experimental configurations enabled an assessment of the influence of the transmission system architecture on the characteristics of electromagnetic emissions and the associated risk of unauthorized information disclosure.

The video codec and compression parameters were not treated as controlled experimental variables in the present study. The objective was not to recover the compressed digital bitstream transmitted through the UAV radio link, but to investigate compromising electromagnetic emissions generated by the operator controller after reception of the video stream. At this stage, the controller performs stream decoding, frame synchronization, image rendering, and visual presentation.

Video compression may indirectly affect the recorded emissions by changing the statistical properties of the encoded stream and the computational activity of the decoding subsystem. In particular, stronger compression and coefficient quantization may reduce fine image details before decoding and may consequently affect the amount of recoverable information. A systematic comparison of codec types, compression ratios, bitrates, and quantization parameters was outside the scope of the present study and should be addressed in future investigations.

### 4.4. Transmitted Test Images

To ensure the repeatability of the experiment and to identify the relationship between the characteristics of electromagnetic emissions and the information content of the image, a set of still test images with varied geometric structures and colors was used. The test images used, shown in Figure 9, are:

– A black triangle on a white background;
– A black pentagon on a white background;
– An image containing numbers (“infiltration map”) in contrasting black and white;
– A color model of a 3D robot.

The selection of the test images was deliberate and intended to facilitate the analysis of the influence of various image characteristics on electromagnetic emissions. Simple geometric patterns enabled the assessment of the effects of distinct contrast transitions and uncomplicated spatial structures on the generated signals. Images containing textual and numerical elements were used to evaluate the potential for the disclosure of symbolic information, whereas a multicolored three-dimensional model enabled the analysis of emissions associated with more complex visual features, including brightness gradients, textures, and color variations.

The use of controlled test patterns rather than natural aerial imagery was intentional, as it enabled the influence of contrast, spatial structure, and image complexity to be assessed under repeatable conditions. Realistic aerial images containing objects with different sizes and contrast levels will be considered in future studies as an additional validation stage.

Each test image was presented to the UAV camera for a predetermined period of time, ensuring stable acquisition of electromagnetic signals and enabling multiple measurement repetitions under identical experimental conditions.

All test images were transmitted under identical measurement conditions and using the same measurement path configuration. Within a given measurement series, the only variable was the visual content captured by the UAV camera. This approach enabled us to investigate the influence of the visual content on the characteristics of the recorded electromagnetic emissions and on the feasibility of subsequent image reconstruction.

A comparison of the frequency characteristics obtained for the individual test images showed that changes in image content did not result in significant shifts of the dominant spectral components or in unambiguous amplitude variations that could be identified through broadband spectrum analysis. The differences observed between the spectra corresponding to different image contents were relatively small and, in many cases, comparable to the natural variability of the tested system’s signal.

These results indicate that information related to image content does not necessarily manifest itself through the emergence of new, readily identifiable frequency components. Instead, such information may be encoded in temporal, amplitude, and synchronization variations within the existing emission components of the signal. For this reason, the subsequent analysis focused not only on conventional spectral comparisons but primarily on time-domain signal analysis and rasterization, which enabled the reconstruction of the spatial structure of the transmitted image.

### 4.5. Measurement Procedure and Signal Analysis

The measurement procedure consisted of recording electromagnetic emissions generated during the transmission of individual test images for both UAV platforms and for each experimental configuration. The procedure comprised the following stages:

1. Preparation of the test image;
2. Positioning of the UAV with respect to the test target;
3. Initiation of image transmission to the operator controller;
4. Acquisition of electromagnetic emissions within the frequency range of 1 MHz–12 GHz;
5. Measurements conducted for both vertical and horizontal antenna polarizations;
6. Storage and post-processing of the recorded measurement data.

The acquired signals were subjected to comparative analysis to identify electromagnetic components correlated with the transmitted visual content. The analysis included the identification of characteristic spectral components, the evaluation of signal amplitude variations, and the investigation of relationships between image features and electromagnetic emission characteristics. Particular attention was devoted to identifying signals that could enable the partial reconstruction of visual information and to assessing the influence of transmission system architecture on the susceptibility of the investigated UAV platforms to electromagnetic eavesdropping.

The electromagnetic signal recorded by the measurement receiver can be represented as:

(1) $$r_{signal}(t)=s_{e}(t)+n(t),$$

where $s_{e}(t)$ denotes the electromagnetic-emission component potentially related to the processed visual information and n(t) represents noise and unrelated electromagnetic disturbances.

After selecting the frequency region used for reconstruction, the signal can be expressed as:

(2) $$r_{B}(t)=F^{-1}\{H_{B}(f)R(f)\},$$

where R(f) is the Fourier transform of the recorded signal and $H_{B}(f)$ denotes the frequency-domain selection function.

The amplitude envelope used for raster reconstruction is then obtained as:

(3) $$a(t)=D\{r_{B}(t)\},$$

where $D\{r_{B}(t)\}$ denotes the amplitude-detection operation.

Following temporal synchronization, consecutive samples of the detected signal are mapped onto a two-dimensional raster according to

(4) $$I_{0}(x,y)=a\left(t_{0}+yT_{H}+x\Delta t\right),$$

with

(5) $$\Delta t=\frac{T_{H}}{N_{x}},$$

where $T_{H}$ is the estimated line period, $N_{x}$ is the number of samples assigned to one raster line, $t_{0}$ is the synchronization offset, and $I_{0}(x,y)$ denotes the raw reconstructed image. The resulting raster is subsequently subjected to normalization, noise reduction, contrast enhancement, and edge-enhancement procedures described below.

The relationship between electromagnetic emissions and the transmitted image content was analyzed using two complementary approaches. First, the frequency characteristics of the signals recorded for the individual test images were compared. This analysis revealed no significant or unambiguous changes in the positions of the dominant spectral components that could be directly attributed to variations in image content. The amplitude differences observed between the individual image variants were also small and did not provide a sufficient basis for unequivocally associating a specific frequency component with a particular image feature.

To quantitatively assess the quality of the resulting reconstructions, the reconstructed images were compared with their corresponding reference images. Before comparison, the images were size normalized, spatially aligned, and, for luminance analysis, converted to grayscale. The Structural Similarity Index (SSIM), Peak Signal-to-Noise Ratio (PSNR), and Pearson’s correlation coefficient (r) were used for evaluation.

The SSIM coefficient is used to evaluate the structural similarity of the reference image x_i_ and the reconstructed image y. The measure takes into account the differences in brightness, contrast and local image structure and is defined as [15]:

(6) $$SSIM(x,y)=\frac{\left(2\mu_{x}\mu_{y}+C_{1})(2\sigma_{xy}+C_{2}\right)}{\left(\mu_{x}^{2}+\mu_{y}^{2}+C_{1})(\sigma_{x}^{2}+\sigma_{y}^{2}+C_{2}\right)}$$

where $\mu_{x},\mu_{y}\;$ mean the average brightness values of the reference and reconstructed images, respectively. $\sigma_{x}^{2},\sigma_{y}^{2}\;$denote the variances of pixel values, where, $\sigma_{xy}$ is the covariance between the pixel values of the two images, and $C_{1}$ and $C_{2}$ are stabilization constants introduced to avoid numerical instability. These constants are defined as follows [15]:

(7) $$C_{1}=(K_{1}L{)}^{2}\;C_{2}=(K_{2}L{)}^{2}$$

where L denotes the dynamic range of pixel values. For 8-bit images, L=255, while the standard values are $K_{1}=0.01$ and $K_{2}=0.03$.

An SSIM value of 1 indicates full structural consistency of the images, while decreasing the coefficient value indicates an increase in the differences between the reconstructed and reference images.

PSNR is a measure of the level of distortion in the reconstructed image relative to the reference image. Calculating PSNR requires first determining the Mean Squared Error (MSE) [15,16]:

(8) $$MSE=\frac{1}{MN}\sum_{i=1}^{M}\sum_{j=1}^{N}\left[I(i,j)-K(i,j\right){]}^{2}$$

where:

I(i,j)—pixel value of the reference image;

K(i,j)—value of the corresponding pixel of the reconstructed image;

M and N—the number of rows and columns of the image, respectively.

Based on MSE, the PSNR coefficient was determined as [16]:

(9) $$PSNR=10{log}_{10}\left(\frac{MAX_{I}^{2}}{MSE}\right)$$

where $MAX_{I}$ is the maximum possible pixel value of the image. For 8-bit images: $MAX_{I}=255$.

PSNR is expressed in decibels (dB). A higher PSNR value indicates a smaller difference between the pixel values of the reference and reconstructed images. However, it should be noted that, in images reconstructed from electromagnetic emissions, the presence of noise, impulsive interference, and minor synchronization errors can significantly reduce the PSNR value, even when the overall structure of the reconstructed object remains recognizable.

The Pearson correlation coefficient (r) was used to assess the linear relationship between the spatial distribution of pixel values in the reference image and the reconstructed image. The coefficient was determined according to the relationship [17,18]:

(10) $$r=\frac{\sum_{i=1}^{N}(x_{i}-\bar{x})(y_{i}-\bar{y})}{\sqrt{\sum_{i=1}^{N}(x_{i}-\bar{x}{)}^{2}\sum_{i=1}^{N}(y_{i}-\bar{y}{)}^{2}}}$$

where:

$x_{i}$—value of the i-th pixel of the reference image;

$y_{i}$—value of the corresponding pixel of the reconstructed image;

$\bar{x}$—average pixel value of the reference image;

$\bar{y}$—average pixel value of the reconstructed image;

N—total number of pixels compared.

The coefficient takes the values:

(11) −1≤r≤1

A value close to 1 indicates a strong positive correlation between the images, a value close to 0 indicates no linear relationship, and a value close to −1 indicates a strong negative correlation. In the analyzed case, positive r values indicate the preservation of the relationship between the spatial structure of the reference image and the reconstructed image.

For images containing simple geometric shapes, the Intersection over Union (IoU) metric was additionally employed. It quantifies the degree of overlap between the area of the reconstructed object and the corresponding area in the reference image [19]:

(12) $$IoU=\frac{|A\cap B|}{|A\cup B|}$$

where:

A—object area in the reference image;

B—object area in the reconstructed image;

A∩B—common part of both areas;

A∪B—the sum of the areas occupied by both objects.

The range of coefficient values is:

(13) 0≤IoU≤1

An IoU value of 1 indicates perfect overlap between the reconstructed and reference objects, whereas an IoU value of 0 indicates no overlap.

The second measure used to assess geometric similarity was the Dice Similarity Coefficient (DSC), defined as [20]:

(14) $$DSC=\frac{2|A\cap B|}{|A|+|B|}$$

where A and B denote the areas of the reference object and the reconstructed object, respectively.

The Dice Similarity Coefficient can also be expressed in terms of the numbers of true positive (TP), false positive (FP), and false negative (FN) pixels as follows:

(15) $$DSC=\frac{2TP}{2TP+FP+FN}$$

where:

TP (True Positive)—number of pixels correctly classified as belonging to the object;

FP (False Positive)—the number of pixels incorrectly classified as belonging to the object;

FN (False Negative)—number of object pixels that were not detected.

The Dice coefficient takes the following values: 0≤DSC≤1, where values close to 1 indicate a high degree of agreement between the shape and position of the reconstructed object and those of the reference object.

The SSIM metric was used to determine the degree to which the spatial structure of the image was preserved, taking into account variations in luminance, contrast, and local image structure. An SSIM value close to 1 indicates a high degree of structural similarity between the reconstructed and reference images. PSNR was used as a measure of the pixel-level error between the images, with higher values indicating lower levels of distortion in the reconstructed image. The Pearson correlation coefficient was employed to assess the linear relationship between the spatial distributions of pixel intensities in the two images; positive values close to 1 indicate a high degree of similarity in their spatial structures. For simple geometric shapes, namely triangles and pentagons, the Intersection over Union (IoU) and Dice Similarity Coefficient (DSC) metrics were additionally applied following segmentation. These metrics enabled the assessment of the degree of overlap between the reconstructed objects and their reference counterparts, thereby providing a quantitative measure of the preservation of shape information regardless of the noise present in the image.

To quantitatively assess the quality of the reconstructed images, each reconstructed image was compared with its corresponding reference image. Prior to the analysis, the images were normalized in size, spatially aligned, and, for luminance-based analysis, converted to grayscale. Three image quality metrics were used: the Structural Similarity Index (SSIM), Peak Signal-to-Noise Ratio (PSNR), and Pearson correlation coefficient (r). For simple geometric shapes, namely a triangle and a pentagon, the Intersection over Union (IoU) and Dice Similarity Coefficient (DSC) metrics were additionally applied, enabling an assessment of the extent to which the geometry of the reconstructed object was preserved.

## 5. Research Results

This section presents the experimental results obtained for the Autel EVO II Pro and DJI Mavic 3 platforms under the two investigated image-presentation configurations. The results include broadband electromagnetic-emission characteristics and the reconstruction of selected visual content. Two unmanned aerial systems were analyzed: the Autel EVO II Pro and the DJI Mavic 3. Each platform was investigated in two operating configurations: a standard configuration involving only the UAV controller and an extended configuration incorporating an additional television receiver connected via an HDMI interface.

The results were presented in the form of electric field strength versus frequency characteristics obtained over the frequency range from 1 MHz to 12 GHz. For comparative purposes, the measured emissions of each system were analyzed with respect to the electromagnetic background level recorded inside the anechoic chamber.

### 5.1. Radiated Emission Characteristics of the Autel EVO II Pro Platform

The first stage of the investigation focused on electromagnetic emissions generated by the Autel EVO II Pro platform operating in its standard configuration, consisting solely of the operator controller. The measurement results, presented in Figure 10, demonstrate that the recorded emission levels significantly exceed the electromagnetic background of the anechoic chamber over a broad frequency range, particularly for frequencies above 100 MHz.

The most prominent spectral components were observed between approximately 1 GHz and several gigahertz, where numerous local maxima in electric field strength were identified. The highest emission levels occurred in the upper portion of this frequency range and exceeded 100 dBµV/m, indicating intensive electromagnetic activity associated with the operation of the platform’s communication and signal-processing subsystems.

In the low-frequency region below approximately 100 MHz, the recorded emission levels remained relatively stable and only marginally exceeded the electromagnetic background measured within the anechoic chamber. As the frequency increased, a gradual growth in emission intensity was observed, accompanied by the emergence of distinct narrowband spectral components. These components may be attributed to the operation of internal clock generation circuits, digital data buses, and communication subsystems responsible for signal processing and transmission within the UAV controller.

A substantial modification of the emission characteristics was observed when the system configuration was extended by incorporating an external television receiver connected through an HDMI interface, as illustrated in Figure 11. Compared with the baseline configuration, this arrangement resulted in a marked increase in radiated emission levels over nearly the entire frequency spectrum under investigation. The most significant differences were observed above 1 GHz, where both the density of spectral components and their amplitudes increased considerably.

Furthermore, the measured spectra exhibited a more complex structure, suggesting the presence of additional electromagnetic emission sources associated with high-bandwidth digital video transmission. This behavior is consistent with the characteristics of the HDMI standard, which relies on high-speed TMDS differential signaling and may generate harmonics as well as other secondary spectral components.

These results indicate that the inclusion of additional image-processing and display elements increases the number of potential sources of compromising emanations and may therefore elevate the risk of information leakage through electromagnetic eavesdropping techniques.

### 5.2. Radiated Emission Characteristics of the DJI Mavic 3 Platform

Similar measurements were conducted for the DJI Mavic 3 platform. In the baseline configuration, comprising only the UAV controller, the recorded results (Figure 12) demonstrated the presence of significant radiated electromagnetic emissions. The overall emission characteristics were partially consistent with those observed for the Autel EVO II Pro platform. In particular, elevated electric field strength levels were identified at frequencies exceeding approximately 1 GHz, together with numerous distinct local spectral peaks. These observations indicate the presence of multiple electromagnetic emission sources associated with the operation of the controller’s digital signal processing and communication subsystems.

Compared with the Autel EVO II Pro platform, the DJI Mavic 3 system exhibited more pronounced amplitude variations in the higher frequency regions of the analyzed spectrum. Strong narrowband spectral components were identified, reaching levels above 100 dBµV/m, particularly in the several-gigahertz frequency range. Such behavior may be associated with intensive activity of the communication, signal-processing, and image-rendering subsystems.

As observed for the Autel platform, the extended configuration incorporating an external television receiver connected via an HDMI interface resulted in a substantial increase in radiated electromagnetic emission levels (Figure 13). An increase in both the number of spectral peaks and their amplitudes was observed, especially at frequencies exceeding 1 GHz. Moreover, the measured spectrum exhibited a more complex structure, characterized by a larger number of impulsive and harmonic components. These observations suggest the presence of additional electromagnetic emission sources associated with high-bandwidth digital image transmission.

Another noteworthy observation was the increase in emission levels within the lower-frequency region of the analyzed spectrum. Compared with the standard configuration, the extended configuration exhibited a noticeably elevated electromagnetic background level. This phenomenon may be attributed to the operation of additional signal-processing and image-display subsystems introduced into the transmission chain through the incorporation of the external television receiver and HDMI interface.

### 5.3. Comparative Analysis of UAV Platforms

Figure 14 shows a comparison of the electromagnetic emission characteristics of both platforms, which indicates both similarities and differences in the spectral structure of the generated signals. For both systems, an increase in electromagnetic activity was noticeable with increasing frequency, especially in the range from approximately 1 GHz to 10 GHz, where the highest emission levels were observed.

In the standard configurations, the DJI Mavic 3 system exhibited a greater number of pronounced high-amplitude narrowband spectral components, whereas the Autel EVO II Pro platform was characterized by a more uniform spectral response with distinct frequency dominants. In both systems, the inclusion of an external television receiver connected via an HDMI interface resulted in a substantial increase in radiated electromagnetic emission levels, particularly at frequencies above 1 GHz.

These results suggest that the incorporation of high-bandwidth digital image transmission interfaces may significantly increase the number of potential sources of compromising emanations and consequently elevate the risk of information leakage through electromagnetic eavesdropping techniques.

### 5.4. Significance of the Results in the Context of Compromising Emanations

The obtained results confirm the presence of characteristic radiated electromagnetic emissions generated by the investigated UAV systems during image transmission. The recorded spectral components, which significantly exceeded the electromagnetic background level measured within the anechoic chamber, indicate the existence of electromagnetic signals potentially associated with visual data processing.

Of particular importance is the presence of strong spectral components in the several-gigahertz frequency range, corresponding to the operating frequencies of modern wireless communication systems and high-speed digital interfaces. The results demonstrate that the architecture of the image transmission chain has a significant impact on both the level and spectral characteristics of electromagnetic emissions and may therefore influence the susceptibility of UAV systems to electromagnetic reconnaissance and eavesdropping techniques.

These findings provide a foundation for further research aimed at identifying relationships between electromagnetic emission characteristics and transmitted image content, particularly in the context of reconstructing visual information from compromising emanations and assessing the electromagnetic security of UAV-based image transmission systems.

### 5.5. Possibility of Reconstructing the Transmitted Image

A significant element of the research was the evaluation of the possibility of reconstructing visual information on the basis of recorded compromising emanations generated during image transmission by the UAV system. The analysis included attempts to reconstruct various classes of test images characterized by different spatial structures, levels of detail, and contrast.

The recorded electromagnetic signals were processed to enable the visualization of spatial structures potentially correlated with the transmitted image content. The obtained results indicate that it was possible to reconstruct characteristic geometric features and selected structural information from the test images; however, the quality of the reconstructed images strongly depended on the complexity of the analyzed scene.

An important observation arising from the conducted research is that the ability to reconstruct visual information was not directly associated with the presence of pronounced changes in the broadband spectral characteristics of the signal following changes in the transmitted image content. For the individual test images, the positions of the dominant spectral components remained largely unchanged, while the observed differences in amplitude were relatively small.

This means that conventional amplitude spectrum analysis is not sufficient to directly determine the content of the transmitted image. The relevant information is contained primarily in the temporal structure of the recorded emissions and in amplitude variations synchronized with the generation and display of successive image elements. By appropriately selecting the horizontal and vertical synchronization parameters and rasterizing the signal, it was possible to map these variations into a two-dimensional spatial structure.

From the perspective of compromising electromagnetic emanation analysis, this finding is important because it indicates that the absence of clear changes in the global signal spectrum does not imply the absence of information correlated with the transmitted visual content. Such information may remain embedded within the temporal structure of apparently similar electromagnetic components and become observable only after the application of appropriate signal acquisition, synchronization, and rasterization techniques.

Images obtained through the rasterization of compromising emanations were characterized by substantial quality degradation resulting from a limited signal-to-noise ratio, synchronization instability, and the presence of electromagnetic interference originating from both the UAV system and the measurement environment. Consequently, the directly reconstructed images contained artifacts in the form of impulse noise, graininess, edge blurring, and local contrast distortions.

To improve image quality, a multistage image-processing algorithm was applied, including:

- Signal amplitude normalization;
- Noise reduction filtering;
- Contrast enhancement;
- Edge sharpening;
- Adaptive image quality enhancement.

In the first stage, the amplitude of the received electromagnetic signal was transformed into the image intensity domain. Let s(t) denote the received electromagnetic signal after amplitude detection, and let I(x,y) denote the corresponding raster image matrix. The normalization process was performed according to the following relationship:

(16) $$I_{N}(x,y)=\frac{I(x,y)-I_{min}}{I_{max}-I_{min}}$$

where:

– $I_{N}(x,y)$—normalized pixel value;
– $I_{min}$—minimum amplitude value;
– $I_{max}$—maximum amplitude value.

Although some of the source test images contained full-color information, the reconstruction procedure employed in this study did not independently recover the red, green, and blue components. The amplitude-detected electromagnetic signal was mapped onto a single scalar image-intensity value I(x,y). Consequently, the reconstructed images represent intensity information and are presented and quantitatively evaluated in grayscale. Independent recovery of individual RGB channels was outside the scope of the present investigation.

The normalization process reduced the influence of dynamic variations in signal amplitude and enhanced the contrast between image objects and the background. Subsequently, impulse noise was suppressed using a median filter, which is particularly effective for eliminating point-like disturbances commonly present in images reconstructed from electromagnetic emissions. The filtered pixel value was calculated according to the following expression:

(17) $$I_{M}(x,y)=median\left\{I_{N}(i,j)\right\}$$

for:

(18) $$\left(i,j)\in \Omega (x,y\right)$$

where:

– $I_{M}(x,y)$—image after median filtering;
– Ω(x,y)—local analysis window (most often 3×3 or 5×5).

The median filter effectively suppressed isolated amplitude noise while preserving edge information to a large extent. To further reduce high-frequency granular artifacts, Gaussian smoothing was subsequently applied. This operation was performed by convolving the image with a Gaussian function, as described by the following equation:

(19) $$I_{G}(x,y)=I_{M}(x,y)*G(x,y)$$

where the Gaussian function is given by the formula:

(20) $$G(x,y)=\frac{1}{2\pi \sigma^{2}}exp\left(-\frac{x^{2}+y^{2}}{2\sigma^{2}}\right)$$

where:

– σ—smoothing parameter;
– $\frac{1}{2\pi \sigma^{2}}$—normalizing factor.

The σ parameter was selected experimentally to maintain the highest possible readability of geometric structures. Adaptive histogram normalization was used to increase the visibility of object boundaries. The brightness transformation was described by the equation:

(21) $$I_{C}(x,y)=\frac{I_{G}(x,y)-\mu }{\sigma_{I}}$$

where:

– μ—average image brightness;
– $\sigma_{I}$—standard deviation of intensity.

In practice, this allowed for increased local contrast between the areas corresponding to the object and the background of the image. To improve the visibility of contours, a Laplace operator was used, responsible for amplifying gradient changes. The operator mask took the form:

(22) $$L=\left[\begin{array}{ccc} 0 & -1 & 0\\ -1 & 4 & -1\\ 0 & -1 & 0 \end{array}\right]$$

The sharpened image was obtained according to the following relationship:

(23) $$I_{S}(x,y)=I_{C}(x,y)+\lambda \nabla^{2}I_{C}(x,y)$$

where:

– λ—sharpening factor;
– $\nabla^{2}$—Laplace operator.

The application of the Laplacian operator enhanced the visibility of geometric object boundaries, particularly in the case of simple shapes such as triangles and pentagons. Furthermore, an image-sharpening procedure based on the difference between the original image and its Gaussian-smoothed counterpart was applied to further improve the clarity of reconstructed features.

(24) $$I_{sharp}=I_{C}+k\left(I_{C}-I_{G}\right)$$

where:

– k—amplification factor.

The method allowed for increasing the clarity of object silhouettes without excessively amplifying noise. The final step involved object segmentation using adaptive thresholding:

(25) $$B(x,y)=\left\{\begin{array}{cc} 1, & I_{sharp}(x,y)>T(x,y)\\ 0, & otherwise \end{array}\right.$$

where T(x,y) means the local brightness threshold.

This processing stage improved the visibility of geometric features and enhanced object recognition in images of higher structural complexity.

The application of the proposed image-processing algorithm resulted in a significant improvement in the quality of images reconstructed from compromising emanations. The most satisfactory results were obtained for high-contrast test images, such as the black triangle and black pentagon presented against a white background, for which clearly identifiable geometric contours were reconstructed. In the case of more complex scenes, including the Infiltration Map image and the 3D robot model, the applied processing techniques primarily improved the recognition of spatial structures and reduced the influence of background noise, thereby increasing the interpretability of the reconstructed visual information [21,22,23,24,25,26,27,28,29,30,31].

#### 5.5.1. Reconstruction of Simple Geometric Figures

The highest reconstruction efficiency was achieved for images containing simple geometric shapes with a high contrast between the object and the background. In the case of a black triangle displayed on a white background, the overall shape, orientation, and position of the object within the image could be reconstructed with a high degree of clarity. Despite the presence of noise and the limited reconstruction resolution, the object boundaries remained sufficiently distinct to enable unambiguous identification of the geometric shape. In particular, the slanted edges of the triangle and the contrast between the object region and the background were reproduced effectively.

A similar effect was observed for the pentagon image, in which the characteristic polygonal shape of the object could also be recognized. However, the greater number of edges and increased geometric complexity resulted in partial contour blurring and a stronger influence of background noise. Nevertheless, the overall geometric structure remained identifiable, confirming the existence of a correlation between electromagnetic emissions and the spatial structure of the transmitted image.

The results obtained, presented in Figure 15 and Figure 16, indicate that images characterized by high contrast and simple spatial features exhibit the greatest susceptibility to reconstruction from compromising emanations. This phenomenon is likely associated with the distinct signal variations generated by abrupt tonal transitions between the bright background and the dark object.

#### 5.5.2. Reconstruction of the Symbolic Image (“Infiltration Map”)

The reconstruction of the image containing numerical and symbolic elements, referred to as the “Infiltration Map,” proved to be considerably more challenging. The results of the reconstruction process are presented in Figure 17 and Figure 18. Despite the relatively high level of noise and interference, it was possible to identify the general spatial arrangement of the information and the presence of structures resembling numbers or symbols.

The reconstructed image preserved the overall spatial organization of information elements corresponding to their arrangement in the original image. However, full readability of individual characters was not achieved, suggesting that effective reconstruction of small textual elements may require more advanced synchronization and signal-processing techniques. Nevertheless, the fact that a portion of the original information structure was preserved indicates the potential for partial disclosure of operational or symbolic information.

From the perspective of information security, this result is particularly significant, as even partial disclosure of data organization, numbering schemes, or image structure may enable the acquisition of valuable intelligence.

#### 5.5.3. Reconstruction of a 3D Robot Image

The greatest reconstruction difficulty was observed for the image depicting a multicolored three-dimensional robot model. Compared with the geometric shapes, the reconstructed image was characterized by higher noise levels and lower sharpness. Despite these limitations, it was possible to recognize the overall silhouette of the object and identify dominant spatial features corresponding to its main contours. The results of this reconstruction are presented in Figure 19 and Figure 20.

The limited reconstruction quality is likely attributable to the significantly greater complexity of the image, including tonal transitions, multiple color components, shading effects, and a large number of local details. Under such conditions, the electromagnetic signals generated by the image-processing system become more complex, reducing the ability to clearly distinguish components correlated with specific image features. Nevertheless, the preservation of a recognizable object silhouette confirms the feasibility of partial image reconstruction even for visually complex scenes.

#### 5.5.4. Quantitative Assessment of Reconstruction Quality

The obtained results were consistent with the observations derived from the qualitative analysis of the reconstructed images. The highest structural similarity was achieved for simple, high-contrast images, particularly the triangle and pentagon test patterns. As the spatial complexity of the image and the number of local variations in brightness and color increased, the reconstruction quality metrics deteriorated. In the case of the image containing numerical information and the 3D model, the overall spatial organization and dominant contours were largely preserved; however, the consistency of fine details was lower.

The quantitative results should be interpreted in light of the specific nature of image reconstruction from electromagnetic emissions. Low PSNR or SSIM values do not necessarily imply a complete absence of useful information, as broadband noise, impulsive interference, and minor synchronization errors can produce significant differences at the pixel level. At the same time, the preservation of an object’s shape, position, and dominant contours may still enable its identification. Therefore, for geometric shapes, the additional use of the IoU and Dice coefficients provides a more reliable assessment of the actual extent of the recovered visual information. Table 1 presents a quantitative assessment of the quality of images reconstructed from unintended electromagnetic emissions, based on the images recorded during the tests.

The relatively low PSNR and SSIM values indicate substantial pixel-level distortion and limited structural similarity; therefore, the reconstructed images should not be interpreted as high-fidelity reproductions of the original content. However, for simple geometric objects, the IoU and Dice values indicate that coarse object geometry, position, and dominant contours may remain preserved despite low pixel-level similarity. Consequently, the demonstrated informational value concerns primarily the recovery of coarse spatial information rather than detailed object identification.

#### 5.5.5. Comparative Analysis of Reconstruction Effectiveness

A comparison of the obtained results indicates that the effectiveness of visual information reconstruction remains strongly dependent on the characteristics of the transmitted image. The highest reconstruction quality was achieved for simple geometric objects with clear contrast, while with increasing scene complexity, the reconstruction quality deteriorated and the influence of interference increased. The research results suggest that, from the perspective of electromagnetic information disclosure, images containing:

- Clear brightness contrasts;
- Large, uniform surfaces;
- Geometric objects with regular edges;
- Large symbols and text elements may be particularly susceptible.

The obtained results confirm the possibility of extracting some visual information based on compromising emanations generated during image transmission by a UAV system. Despite the limited reconstruction quality, the mere fact of being able to recognize shapes, spatial arrangement of objects, or the general content of a scene can pose a significant threat to the security of information processed by unmanned systems.

## 6. Discussion

The conducted research confirmed the presence of compromising emanations generated by commercial unmanned aerial vehicle (UAV) systems during real-time image transmission. The analysis of radiated electromagnetic emissions, together with the performed image reconstruction experiments, demonstrated that both the architecture of the UAV system and the configuration of the transmission chain have a significant influence on the electromagnetic characteristics of the investigated platforms and on the potential for unauthorized information acquisition.

The obtained results indicate that, despite the widespread adoption of modern digital communication technologies and advanced video encoding techniques, contemporary commercial UAV platforms continue to generate unintentional electromagnetic emissions containing components correlated with the processed visual content. This phenomenon is of particular importance in the context of information security, as it enables passive electromagnetic reconnaissance without requiring physical access to the system, interference with its operation, or interception of the wireless communication channel.

### 6.1. Electromagnetic Characteristics of the Tested UAV Platforms

Analysis of the measurement results revealed significant similarities in the radiated emission characteristics of both investigated UAV platforms, while also highlighting noticeable differences in the spectral structure of the generated signals. For both the Autel EVO II Pro and DJI Mavic 3 systems, a substantial increase in electromagnetic emission levels was observed at frequencies above approximately 1 GHz, where the dominant spectral components exhibited the highest electric field strengths.

The experimental data indicate that the primary sources of the observed emissions are subsystems responsible for high-speed digital signal processing, particularly image decoding modules, digital signal processors, graphics rendering units, and high-bandwidth data buses operating within the operator controller. In practice, this indicates that the level of compromising emanations may be more strongly related to the final stage of image processing and presentation than to the radio communication channel used by the UAV for data transmission.

Another noteworthy observation is the relatively low emission level in the lower-frequency ranges and the gradual increase in electromagnetic activity with increasing frequency. This behavior may indicate the dominant influence of modern high-frequency digital circuits, which are characteristic of contemporary mobile devices and multimedia transmission systems.

A comparison of the two platforms indicates that the DJI Mavic 3 exhibited a greater number of distinct high-amplitude narrowband spectral components, whereas the Autel EVO II Pro demonstrated a more uniform spectral response. These differences may result from variations in the implementation of image transmission systems, signal-processing architectures, and visual data encoding and decoding mechanisms.

### 6.2. Influence of Transmission Path Configuration on the Level of Compromising Emanations

One of the most important findings of the conducted research was the demonstration of a significant influence of the image processing chain configuration on the level of electromagnetic emissions. For both analyzed platforms, a substantial increase in electromagnetic activity was observed when the system configuration was extended by incorporating an additional television receiver connected to the controller via an HDMI interface.

In the extended configuration, not only was an increase in emission levels observed, but also a larger number of spectral components appeared, particularly at frequencies exceeding 1 GHz. This effect can be attributed to the operating characteristics of the HDMI interface, which employs high-speed TMDS (Transition-Minimized Differential Signaling) transmission and may generate numerous harmonics and secondary spectral components.

From the perspective of electromagnetic security, this finding is of particular practical importance. It indicates that the use of additional display devices can substantially increase the potential surface for electromagnetic information leakage. In other words, extending the functionality of a UAV system through the use of external monitors or television receivers may unintentionally increase its susceptibility to electromagnetic eavesdropping.

It should be emphasized that the use of external displays is common practice in professional and operational applications, particularly during missions requiring image sharing among multiple operators. The obtained results suggest that such configurations should also be evaluated from the perspective of compromising emanations and electromagnetic security.

From an applied perspective, the presented approach should primarily be interpreted as a method for comparative electromagnetic-susceptibility assessment rather than as an estimate of an operational eavesdropping range. Such measurements may be used to identify configurations that increase the electromagnetic information-leakage surface and to compare alternative architectures of UAV operator stations. In particular, the results indicate that the complete image-presentation chain, including external high-speed interfaces and display devices, should be included in electromagnetic-security assessments.

Scaling the approach toward realistic operational applications requires stepped-distance measurements, active-flight experiments, dynamic video sequences, measurements in the presence of environmental electromagnetic interference, and evaluation of systems equipped with technical protection measures.

### 6.3. Possibility of Reconstructing Visual Information

A particularly important aspect of the conducted research was the assessment of the possibility of reconstructing the transmitted image based on compromising emanations. The obtained results showed that even with limited signal quality it is possible to reproduce the characteristic spatial features of transmitted objects.

The highest reconstruction efficiency was achieved for high-contrast images containing simple geometric figures. Both the triangle and the pentagon remained clearly identifiable despite the presence of noise and limited reconstruction quality. This means that the electromagnetic signals generated by the system show a sufficiently high correlation with the spatial structure of the image to enable recognition of the basic geometric features of the object. Much more difficult was the reconstruction of more complex images, including symbolic structures and multi-colored three-dimensional objects. In the case of the infiltration map, it was possible to partially recognize the data arrangement and the spatial organization of digits, but full identification of text information remained difficult. For the robot model, a recognizable silhouette of the object was obtained, but with limited mapping detail. The obtained results are consistent with observations presented in the literature on emission revealing visualization systems, according to which the effectiveness of reconstruction remains strongly dependent on the image contrast, level of detail and spatial characteristics of the processed scene.

### 6.4. Role of Image Processing Algorithms

A significant aspect of the research was the application of image enhancement and noise reduction procedures to the reconstructed images. The results indicate that appropriately selected signal-processing techniques can substantially improve image clarity and facilitate object identification.

The application of median and Gaussian filtering reduced impulse noise and image graininess, while edge-enhancement procedures improved the visibility of geometric object boundaries. A particularly significant improvement was observed for high-contrast images, for which considerably more stable and clearly distinguishable contours of geometric shapes were obtained.

These results suggest that the actual potential of electromagnetic eavesdropping may be greater than would be inferred from the quality of the raw reconstructed images alone. Even partially degraded images can be subjected to advanced digital image-processing techniques, significantly improving their interpretability.

From the perspective of information security, this implies that the assessment of UAV system vulnerability should not be limited solely to the analysis of electromagnetic emission levels. It should also take into account the capabilities of modern digital signal- and image-processing methods, which may substantially enhance the extraction of useful information from recorded electromagnetic emissions.

### 6.5. Relationship Between Image Content and Electromagnetic Emissions

Comparison of measurements taken for different test images showed that the fundamental frequency structure of the electromagnetic emissions remained essentially unchanged when the image content was changed. The dominant spectral components occurred at similar frequencies, and the differences in their amplitudes were relatively small. Therefore, broadband amplitude-frequency characteristics did not provide a sufficient basis for unequivocally associating specific spectral components with particular visual content.

This observation indicates that the dominant emission frequencies are more strongly related to the physical architecture of the investigated system, the clock frequencies of its digital circuits, data transmission mechanisms, communication interfaces, and the processes involved in image decoding and display than to the content of the displayed scene itself. In contrast, changes in image content affect the instantaneous temporal and amplitude structure of the signal within the same emission components.

This conclusion is supported by the ability to obtain different reconstructed images from signals originating from spectral regions with similar frequency structures. This indicates that the information of interest is not primarily encoded through changes in the positions of characteristic spectral peaks but rather through signal modulation associated with the temporal organization of visual information processing and display.

This result limits the possibility of directly identifying image content solely through classical spectral analysis; however, it also highlights the importance of time-domain analysis, synchronization, and rasterization in the investigation of compromising electromagnetic emissions. Consequently, the absence of a clear relationship between image content and the positions of the dominant spectral components does not preclude the possibility of information recovery. Rather, it indicates a different mechanism through which information is embedded in the recorded emissions.

### 6.6. Influence of Experimental Parameters on Reconstruction

The effectiveness of visual-information reconstruction depends not only on the image content but also on several parameters associated with signal acquisition and processing. One of the most important factors is the signal-to-noise ratio. An increase in the electromagnetic background or receiver noise reduces the contrast between the useful emission component and unrelated disturbances, which may result in poorer synchronization, increased raster artifacts, and reduced reconstruction quality. The controlled anechoic-chamber environment used in the present study intentionally limited this source of variability and therefore represents favorable acquisition conditions.

The selected reconstruction frequency and the amplitude of the received emission are also important. Different frequency components may exhibit different coupling efficiency, propagation characteristics, antenna response, and correlation with the temporal activity of the image-processing circuitry. A higher received amplitude generally improves the signal-to-noise ratio; however, emission amplitude alone does not determine the amount of recoverable information. A strong spectral component that does not preserve sufficiently stable temporal structure may be less useful for reconstruction than a weaker but more coherent component.

Image characteristics constitute another important factor. Large high-contrast structures are less sensitive to limited spatial resolution and synchronization errors, whereas small details, text, and complex local structures require higher temporal stability and effective raster resolution. This behavior is consistent with the experimental results, in which simple geometric objects were reconstructed more reliably than the Infiltration Map and the complex 3D model. A systematic quantitative analysis involving controlled variation of noise level, reconstruction frequency, emission amplitude, image dimensions, measurement distance, and receiver parameters should therefore be included in future studies.

### 6.7. Security Implications and Threat Model

The electromagnetic leakage channel investigated in this study should be considered as one of several possible mechanisms for unauthorized acquisition of UAV visual information. Direct interception of the intended radio communication channel may potentially provide higher-quality data; however, its effectiveness depends on access to the communication protocol and on the applied authentication and encryption mechanisms. Cyber-based attacks may provide broader access to system resources but require an exploitable vulnerability and typically involve active interaction with the target system.

In contrast, compromising electromagnetic emanation analysis is passive and does not require access to the UAV communication protocol or decryption of the transmitted video stream. Its effectiveness is, however, strongly dependent on measurement distance, electromagnetic background, signal-to-noise ratio, system configuration, and synchronization stability. Therefore, the presented approach should not be interpreted as universally more effective than other attack vectors, but rather as an additional passive information-leakage channel that should be considered as part of a broader UAV security assessment.

Spread-spectrum techniques require a distinction between the intentional radio transmission and the unintentional electromagnetic leakage investigated in this study. DSSS, FHSS, or related spread-spectrum techniques applied to the UAV communication link primarily affect the intentional radio waveform. Because the present measurements focus on emissions generated by the operator controller after reception and decoding of the video stream, such radio-link techniques would not necessarily suppress compromising emissions originating from decoding, graphics processing, synchronization, and image display.

Similarly, bit errors and error-correcting coding primarily concern the intentional UAV radio link, whereas the present study focuses on compromising emanations generated after reception and decoding. Residual transmission errors that modify the decoded and displayed image may indirectly affect reconstruction quality; however, controlled variation of BER or packet loss was outside the scope of the present baseline study and should be considered in future work.

A different situation may arise when spectral spreading is intentionally introduced into the internal digital circuitry itself. Spread-spectrum clocking, clock-frequency dithering, or related randomization techniques may redistribute leakage energy over a wider frequency range, reduce distinct narrowband components, and decrease temporal coherence. Such effects may make stable synchronization and raster reconstruction more difficult. The effectiveness of these techniques against UAV compromising emanations was not evaluated in the present study and constitutes an important topic for future investigation.

### 6.8. Scientific Contribution of the Study

It should be emphasized that the primary contribution of this work is not the development of a novel image reconstruction algorithm. The filtering, contrast enhancement, sharpening, and segmentation procedures employed are well-established digital image processing techniques. The originality of this work is primarily experimental and lies in the evaluation of real, commercially available UAV systems with respect to compromising electromagnetic emissions during image transmission.

Specifically, the research enabled a comparison of two commercial UAV platforms, an assessment of the impact of image transmission and display path configurations, including the use of an external HDMI interface and a TV receiver, and an evaluation of the feasibility of partially recovering visual information from recorded electromagnetic emissions. An additional contribution of the study is the comparison of reconstructions obtained for images with varying levels of complexity, together with their quantitative evaluation using image quality and similarity metrics.

The results also demonstrate that an increase in the level of electromagnetic emissions does not necessarily correspond to a proportional increase in the amount of recovered information. Reconstruction performance is also influenced by the temporal characteristics of the signal, synchronization stability, the signal-to-noise ratio, and the spatial structure of the processed image.

### 6.9. Research Limitations

Despite the promising results obtained in this study, several methodological limitations should be acknowledged. First, all measurements were conducted in an anechoic chamber, which allowed environmental electromagnetic interference and multipath propagation effects to be minimized. Although this ensured high measurement repeatability, it also limited the direct transferability of the results to real operational environments.

Second, the investigated UAV platforms remained stationary throughout the experiments and were not operated in flight. Consequently, electromagnetic disturbances associated with propulsion systems, electronic speed controllers, and dynamic platform maneuvers were excluded from the measurements. In practical operating conditions, such factors may influence the spectral characteristics of the recorded electromagnetic emissions.

A further limitation is related to the set of test images used during the experiments. While the selected images represented a range of geometric, symbolic, and visually complex structures, future studies should incorporate realistic video sequences, dynamic scenes, varying illumination conditions, and a wider variety of visual content.

Nevertheless, the obtained results clearly confirm the existence of compromising emanations generated during UAV image transmission and demonstrate the feasibility of partial reconstruction of transmitted visual information. These findings highlight the need for further investigations into the electromagnetic security of UAV systems and their resistance to technical reconnaissance based on electromagnetic eavesdropping.

An additional limitation of the conducted research is the inability to clearly associate individual spectral components with specific image elements or particular electronic components of the controller. A comparison of signals recorded for different visual content revealed only minor differences in their broadband frequency characteristics. The precise localization of physical emission sources and the determination of their contribution to information transfer would require further research, for example, through the use of near-field probes to identify emission sources within the tested device.

At the same time, the ability to reconstruct recognizable images through rasterization confirms that the absence of significant changes in global spectral characteristics does not preclude the presence of information-bearing components. Further research should therefore include more detailed time-frequency and correlation analyses, as well as signal evaluation for a broader range of static and dynamic visual content.

Accordingly, the conclusions of the present study are limited to the investigated UAV platforms, the 1 m measurement distance, the anechoic-chamber environment, and the two analyzed image-presentation configurations.

Furthermore, the measurement distance of 1 m and the absence of additional electromagnetic protection measures at the operator station represent favorable conditions for signal acquisition. These conditions were intentionally selected to obtain a repeatable baseline and to verify the feasibility of visual-information recovery under controlled conditions. Consequently, the present results should not be interpreted as an estimate of the practical interception range of an operational eavesdropping system. Therefore, no operational interception distance is claimed on the basis of the present experiments.

Validation under realistic conditions requires measurements at progressively larger distances, UAV operation during active flight, dynamic video sequences, environmental electromagnetic interference, and operator stations incorporating technical protection measures.

## 7. Conclusions

The conducted research confirmed the presence of compromising emanations generated by commercial unmanned aerial vehicle (UAV) systems during real-time image transmission. Electromagnetic analysis of two popular UAV platforms—the Autel EVO II Pro and the DJI Mavic 3—revealed the presence of characteristic spectral components exceeding the electromagnetic background level of the anechoic chamber. This phenomenon confirms the possibility of identifying signals correlated with the processing and display of visual information.

The conducted studies confirmed the presence of electromagnetic emissions from commercial unmanned aerial vehicle (UAV) systems during the transmission and presentation of visual information. For both tested platforms, characteristic emissions were identified that exceeded the chamber’s electromagnetic background level, with the highest activity observed primarily in the frequency range above approximately 1 GHz.

At the same time, a comparison of measurements taken for different image content showed that changing the displayed information did not significantly alter the position of the dominant spectral components. The amplitude differences between the characteristics obtained for individual images were relatively small and did not allow for the unambiguous assignment of a specific frequency component to a specific image element. This result indicates that broadband spectral analysis alone has limited usefulness in identifying the content of the processed information.

Despite slight differences in the frequency characteristics, it was possible to partially reconstruct the transmitted visual content. This indicates that the information associated with the image is primarily contained in the temporal and amplitude structure of the recorded signal, and that its extraction requires appropriate synchronization, rasterization, and subsequent image processing.

The highest reconstruction efficiency was achieved for simple, high-contrast geometric shapes, such as triangles and pentagons. For more complex images, the overall spatial organization and dominant contours were preserved; however, the level of fine detail was significantly lower. Quantitative evaluation using the SSIM, PSNR, and Pearson correlation coefficients confirmed differences in the reconstruction efficiency among individual image classes. For simple geometric shapes, additional analysis based on the IoU and Dice coefficients enabled a quantitative assessment of the extent to which the geometry of the reconstructed objects was preserved.

Another important research result is the impact of the image presentation path configuration on the level of electromagnetic emissions. Using an external TV receiver connected to the controller via HDMI increased the emission level and the number of observed spectral components. This result indicates that expanding the visualization path may increase the potential surface area for electromagnetic information leakage.

The research findings also indicate the need to consider electromagnetic security issues during the early stages of UAV system design. At present, commercial UAV platforms are optimized primarily for image quality, communication reliability, and operational usability, whereas the mitigation of compromising emanations is rarely considered a primary design objective. Consequently, established EMSEC countermeasures should be evaluated and, where necessary, adapted to the specific architecture of commercial UAV operator stations.

Potential measures for reducing the risk of electromagnetic information leakage include:

- The application of electromagnetic shielding to selected system components;
- The optimization of high-frequency data transmission subsystems;
- The reduction of emissions generated by transmission interfaces, particularly HDMI;
- The implementation of dynamic image-transmission techniques that hinder synchronization and reconstruction processes.

The main contribution of this work is experimental in nature. The study demonstrates that real commercial UAV systems can generate electromagnetic emissions that enable the partial reconstruction of processed visual information, even when changes in image content do not produce readily observable differences in conventional spectral characteristics. The results justify further research, including time-frequency analysis of the emissions, the use of a larger set of test images, the analysis of dynamic video sequences, and the evaluation of UAV operation under real-world conditions.

## Figures and Tables

**Figure 1:**
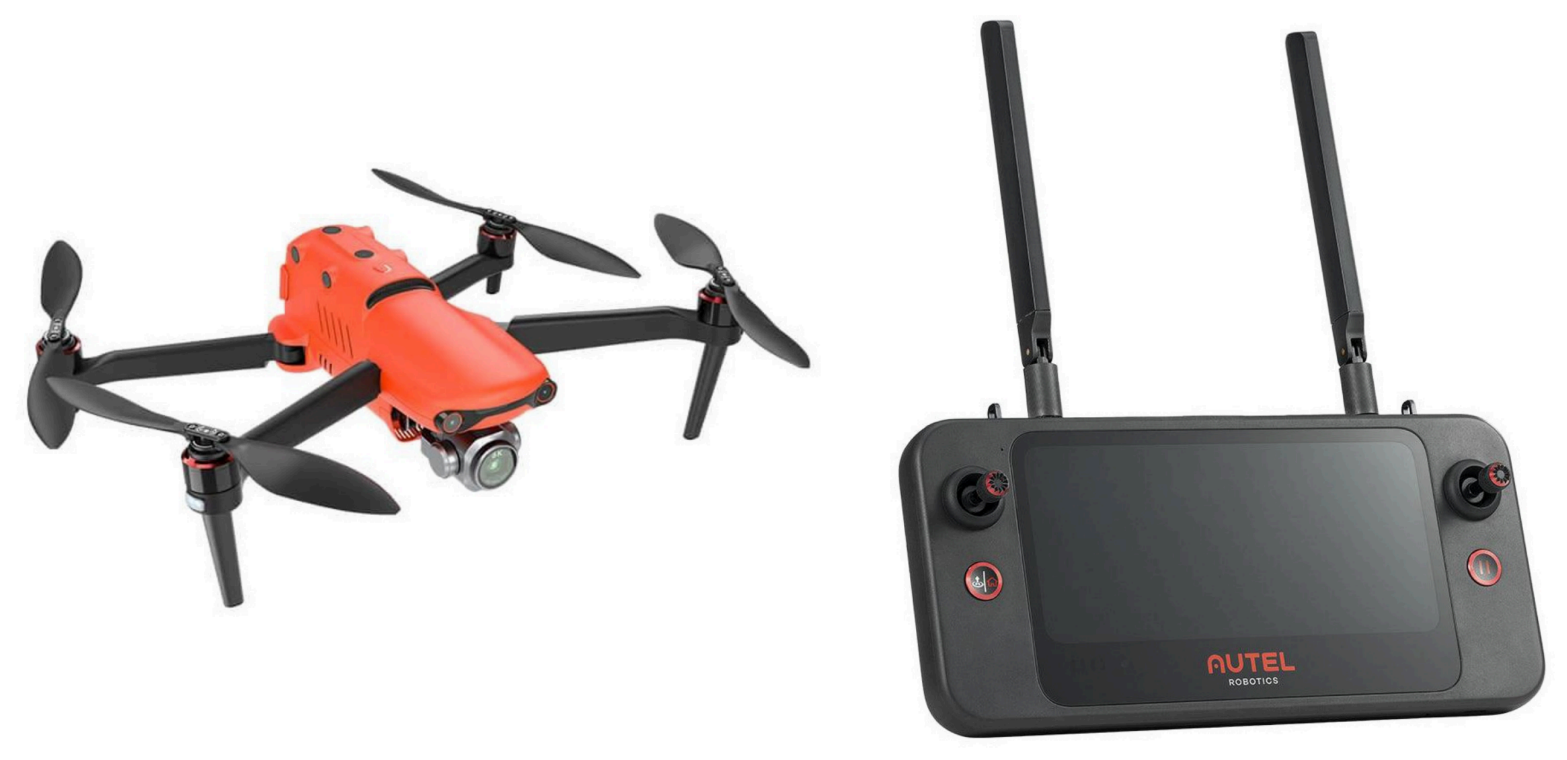
Autel EVO II Pro.

**Figure 2:**
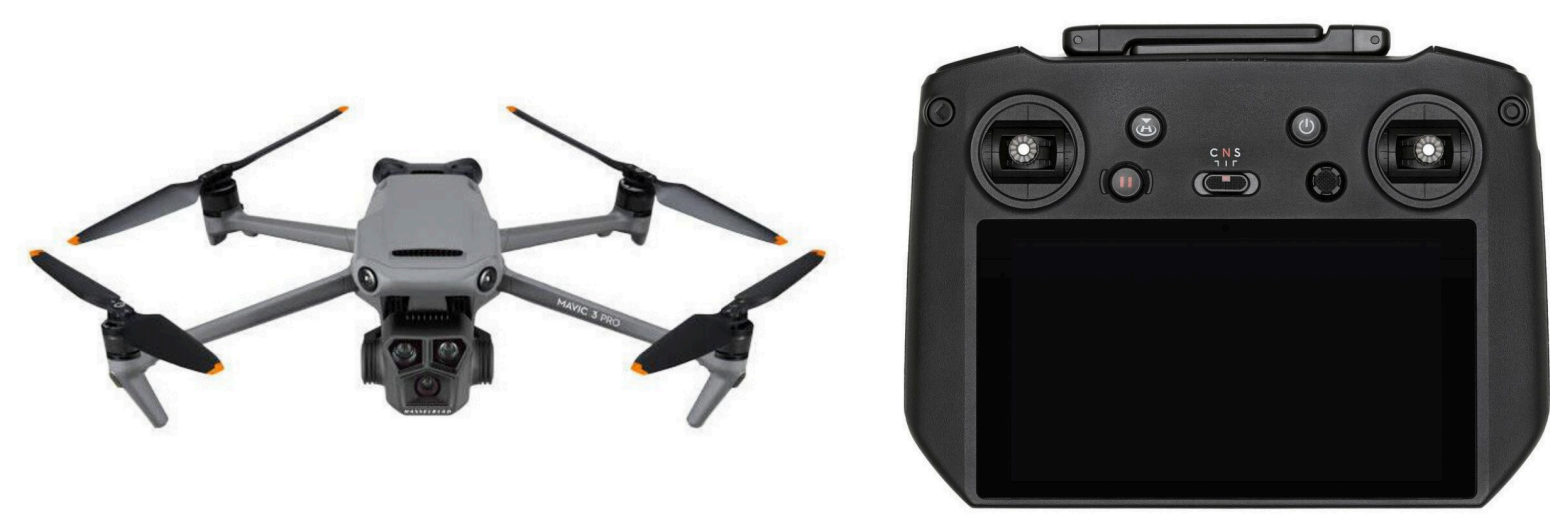
DJI Mavic 3.

**Figure 3:**
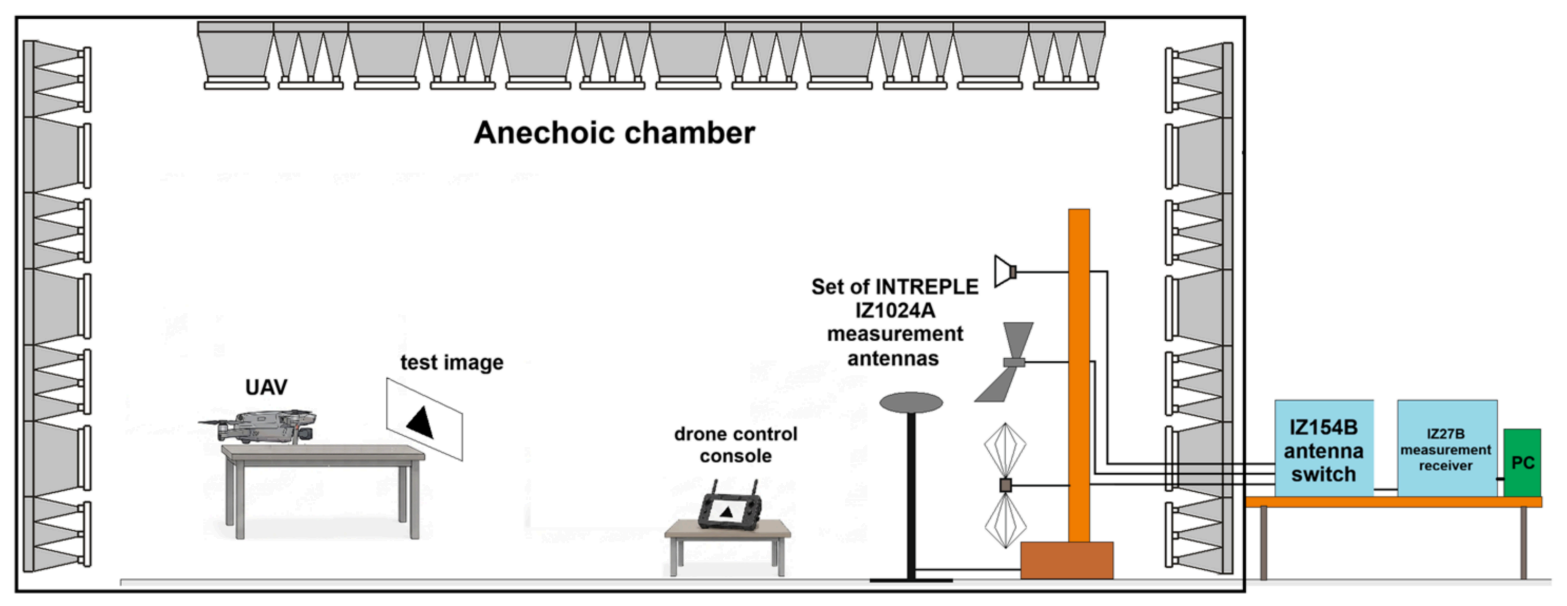
Block diagram of the laboratory stand used for the detection of electromagnetic emissions generated by a UAV during image transmission.

**Figure 4:**
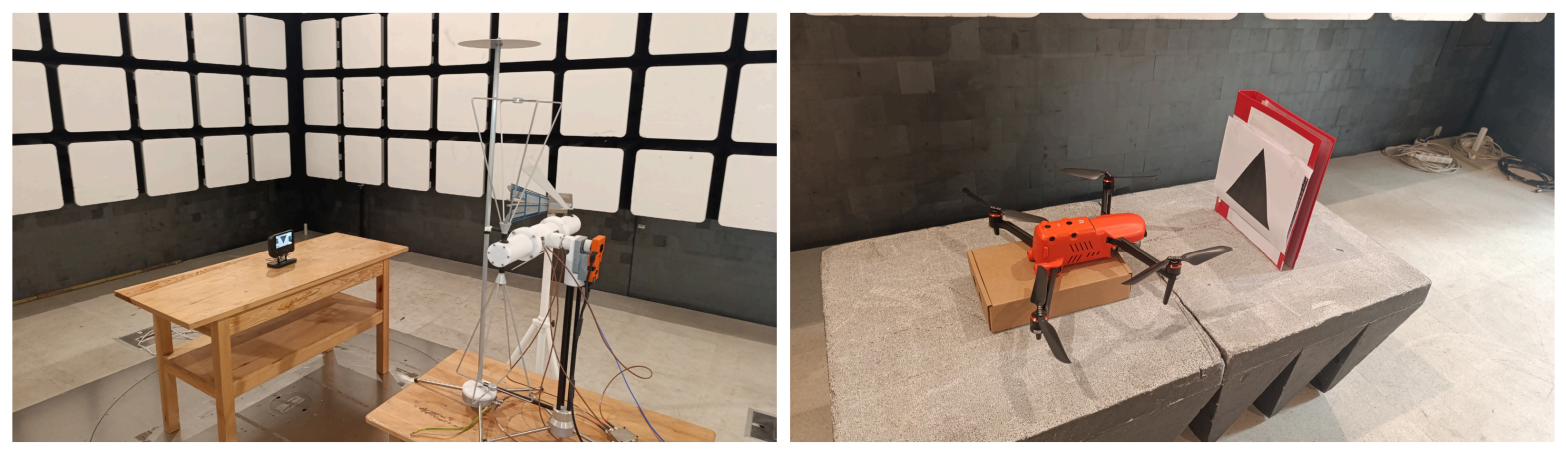
View of the laboratory stand used to measure emissions generated by the unmanned aerial vehicle during testing.

**Figure 5:**
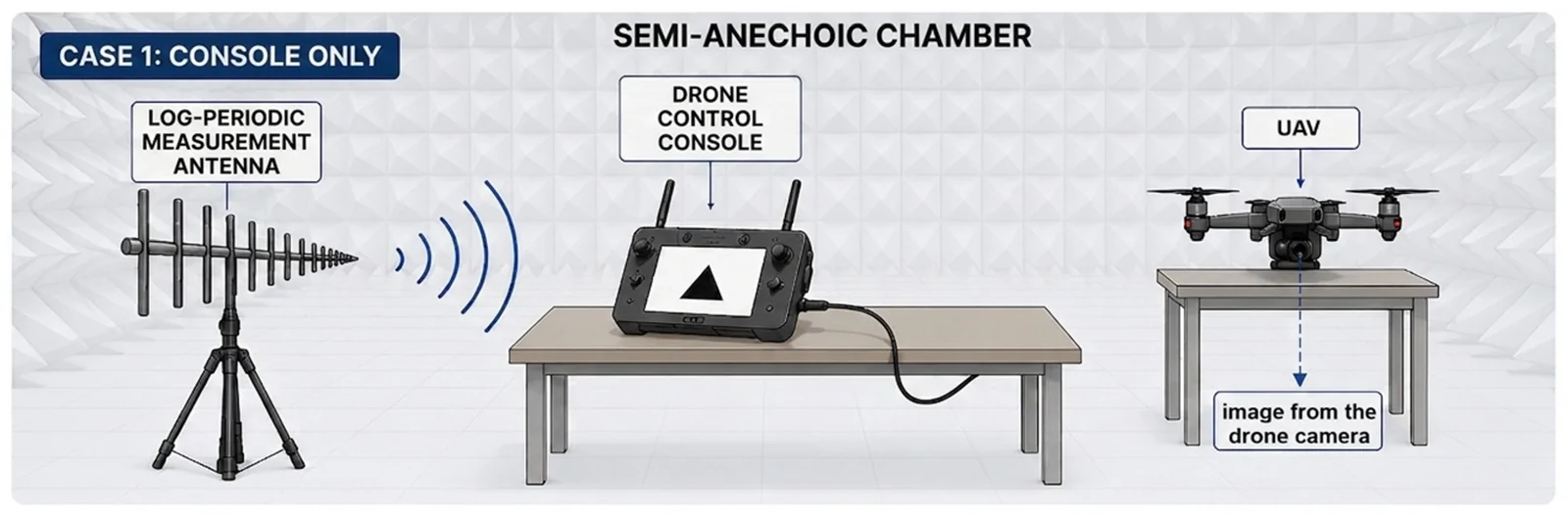
Schematic diagram of the laboratory stand used for investigating compromising emanations generated during UAV image transmission in scenario I—standard image transmission path.

**Figure 6:**
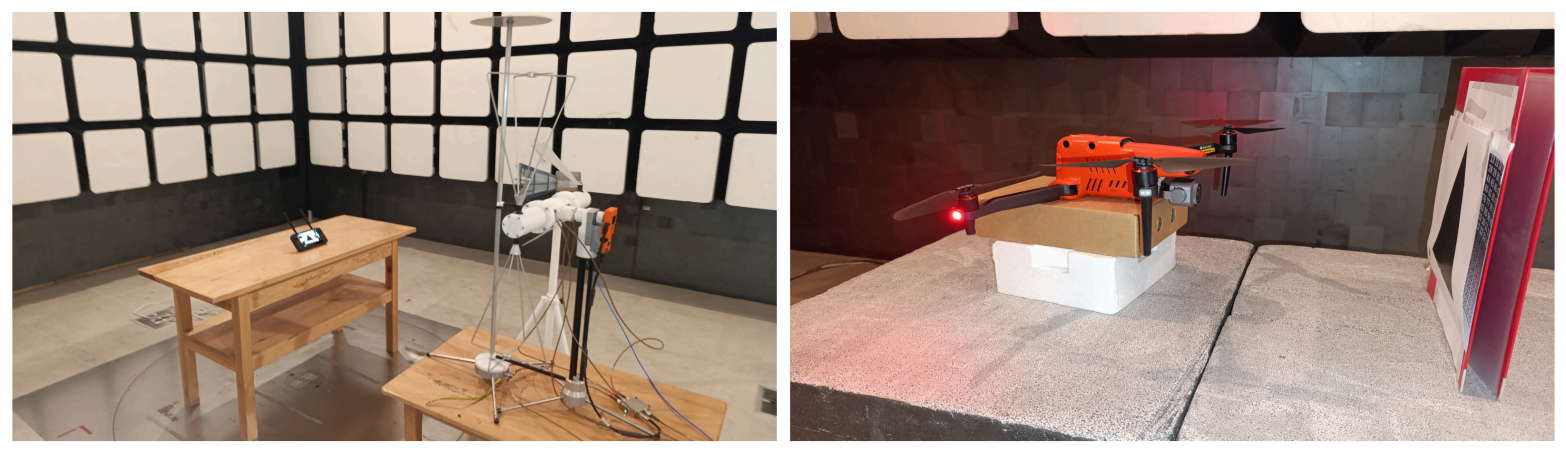
View of the laboratory stand used for investigating compromising emanations generated during UAV image transmission in scenario I—standard image transmission path.

**Figure 7:**
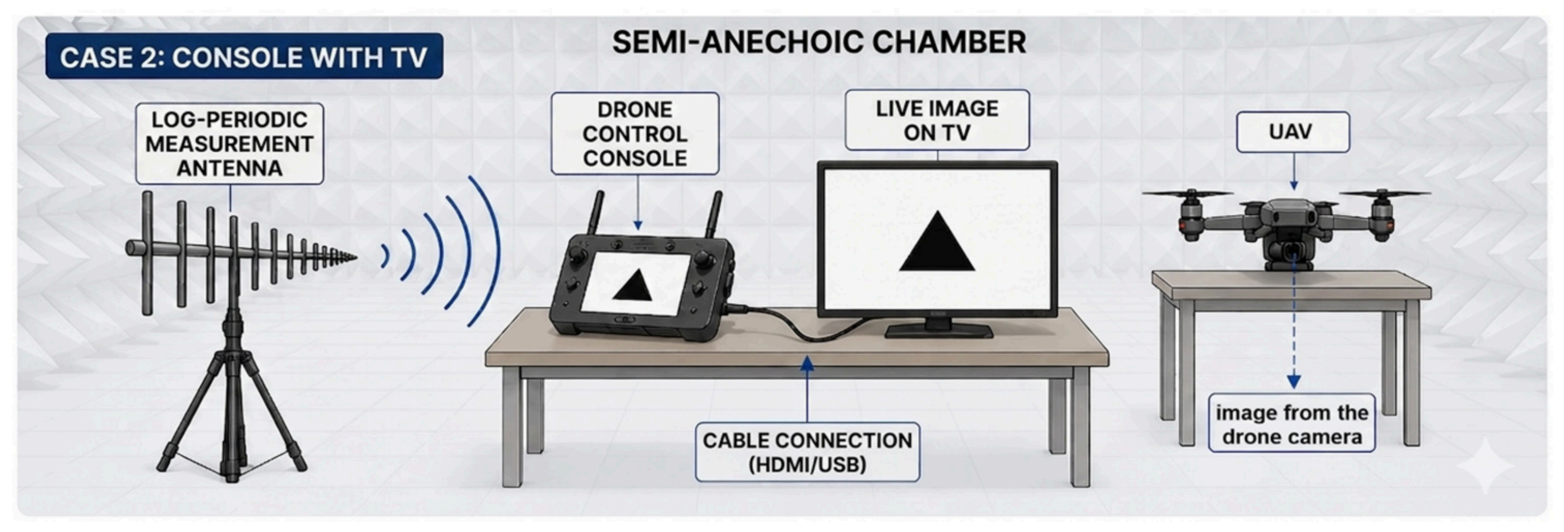
Schematic diagram of the laboratory stand for scenario II—extended image transmission path with an HDMI interface.

**Figure 8:**
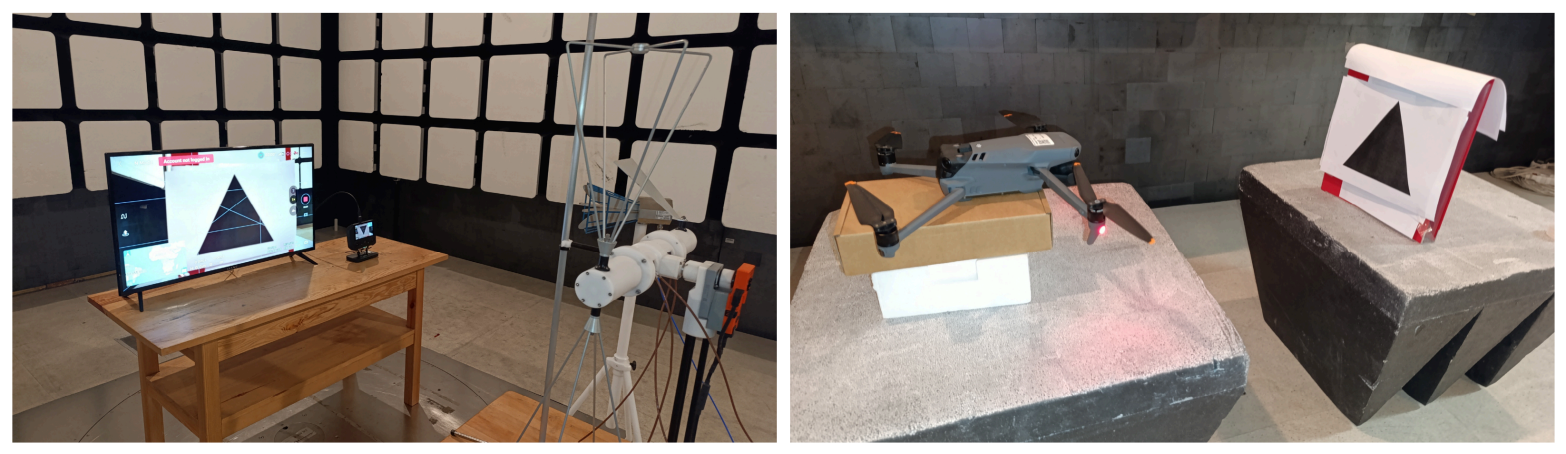
View of the laboratory stand for scenario II—extended image transmission path with an HDMI interface.

**Figure 9:**
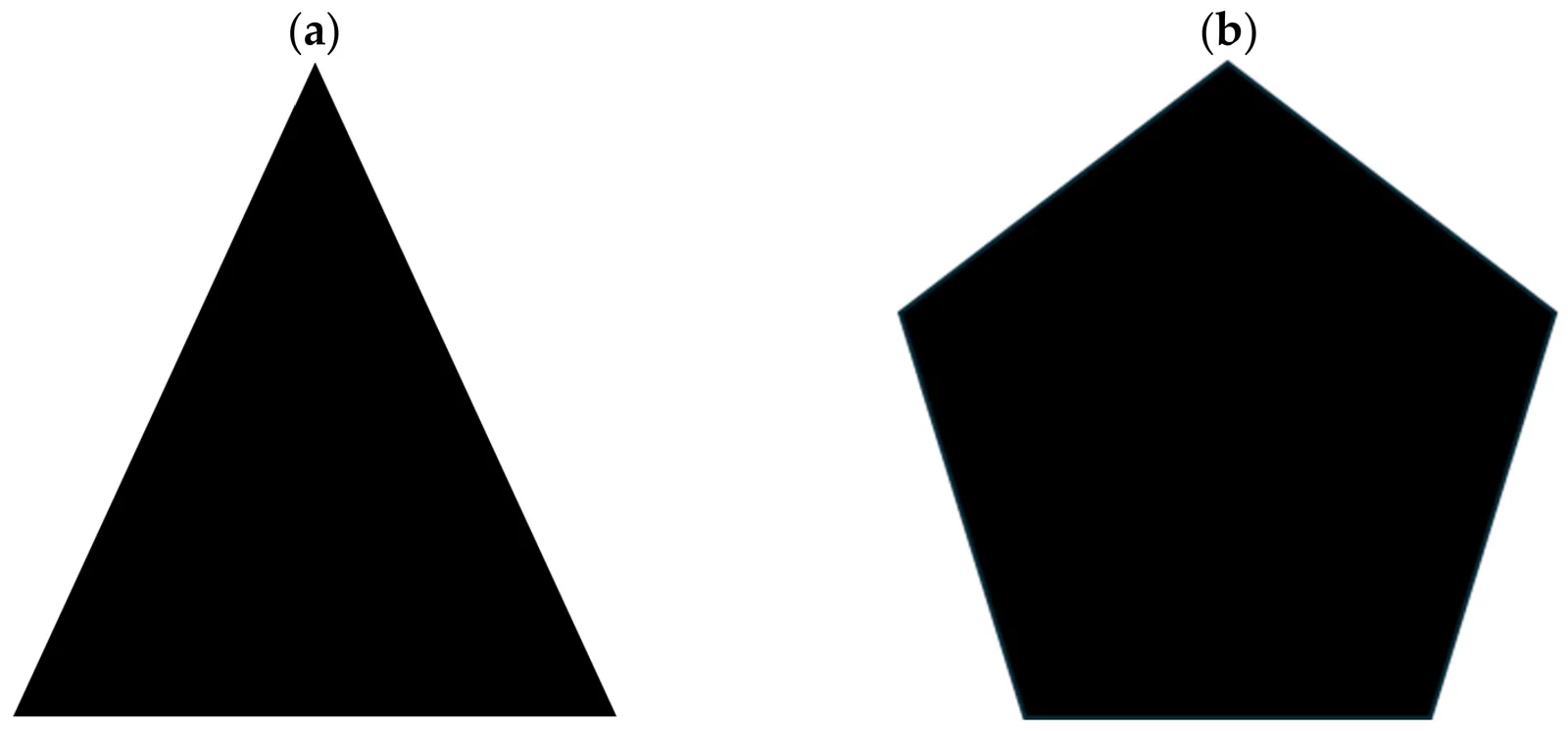

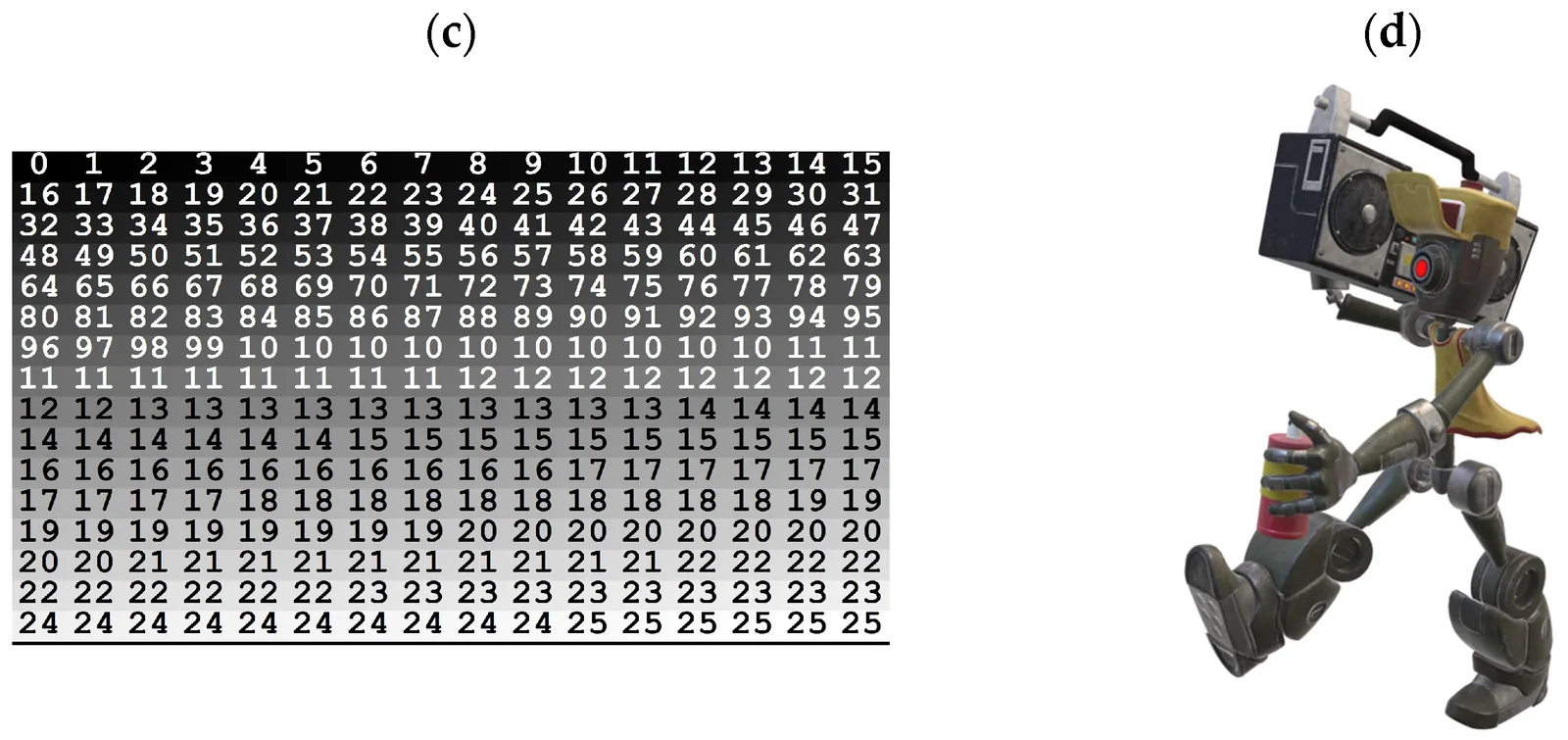
Test images used during the experiments and transmitted between the UAV platform and the operator controller: (**a**) black triangle on a white background; (**b**) black pentagon on a white background; (**c**) high-contrast black-and-white image containing numerical information (“Infiltration Map”); (**d**) color 3D model of a robot.

**Figure 10:**
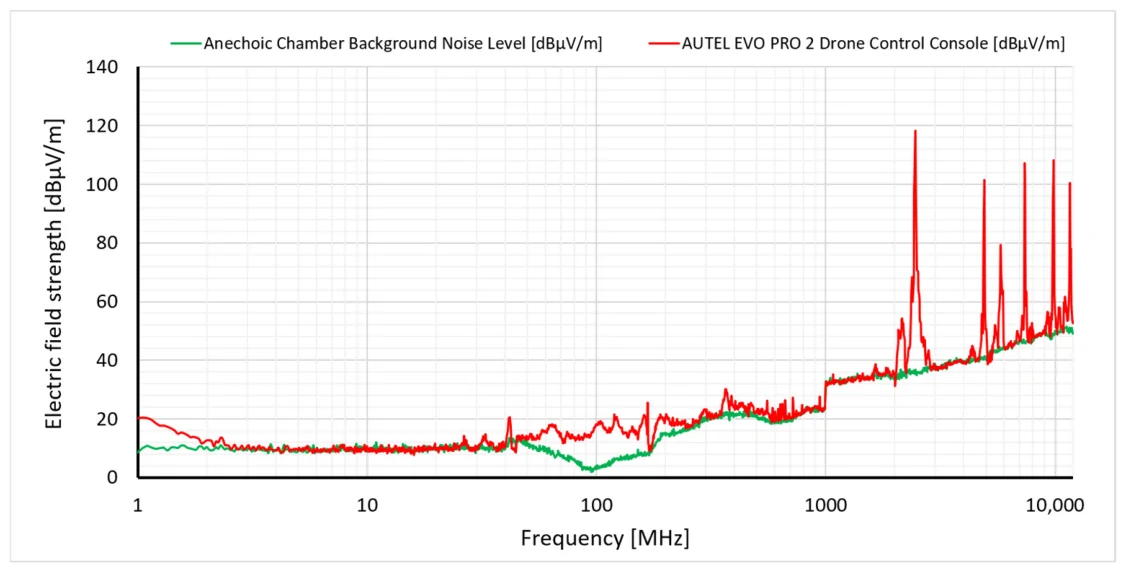
Radiated electromagnetic emission levels generated by the Autel EVO II Pro platform during image transmission to the operator controller in the frequency range from 1 MHz to 12 GHz.

**Figure 11:**
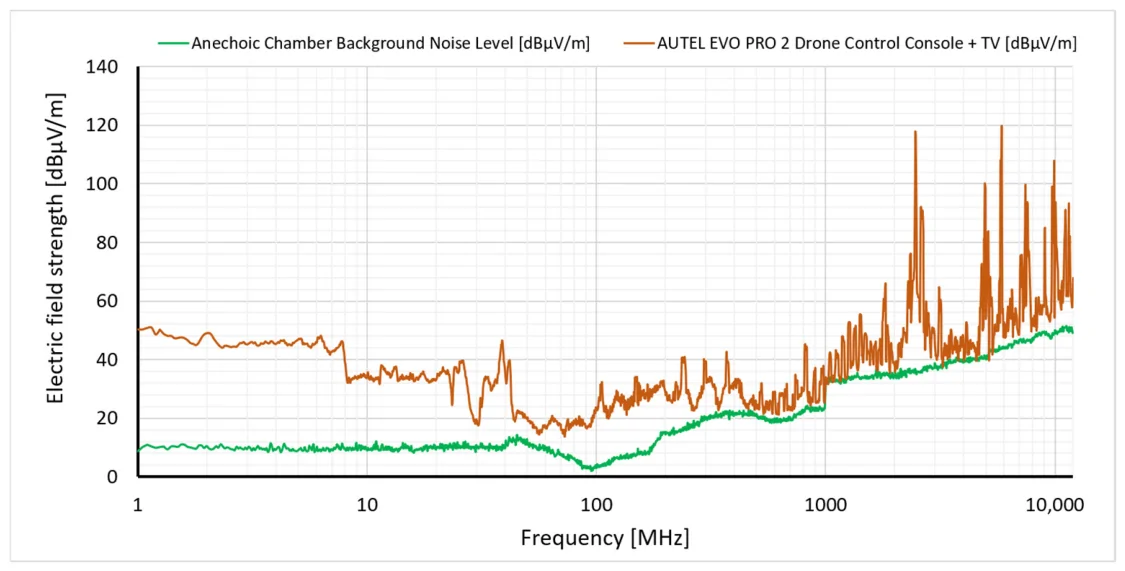
Radiated electromagnetic emission levels generated by the Autel EVO II Pro platform during image transmission to the controller and simultaneous image display on an LED television receiver over the frequency range of 1 MHz–12 GHz.

**Figure 12:**
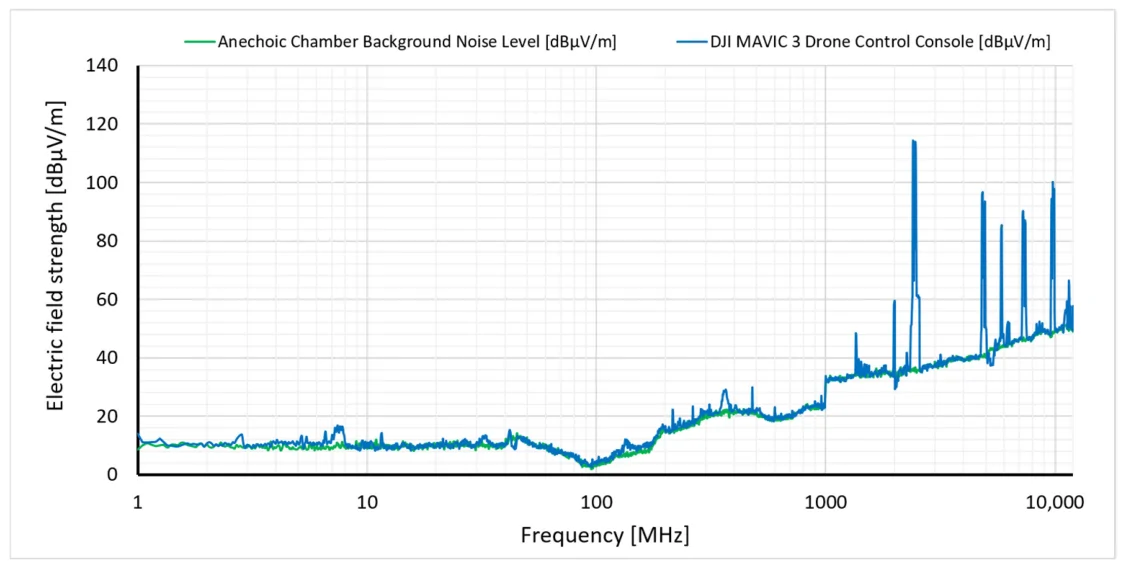
Radiated electromagnetic emission levels generated by the DJI Mavic 3 platform during image transmission to the operator controller in the frequency range from 1 MHz to 12 GHz.

**Figure 13:**
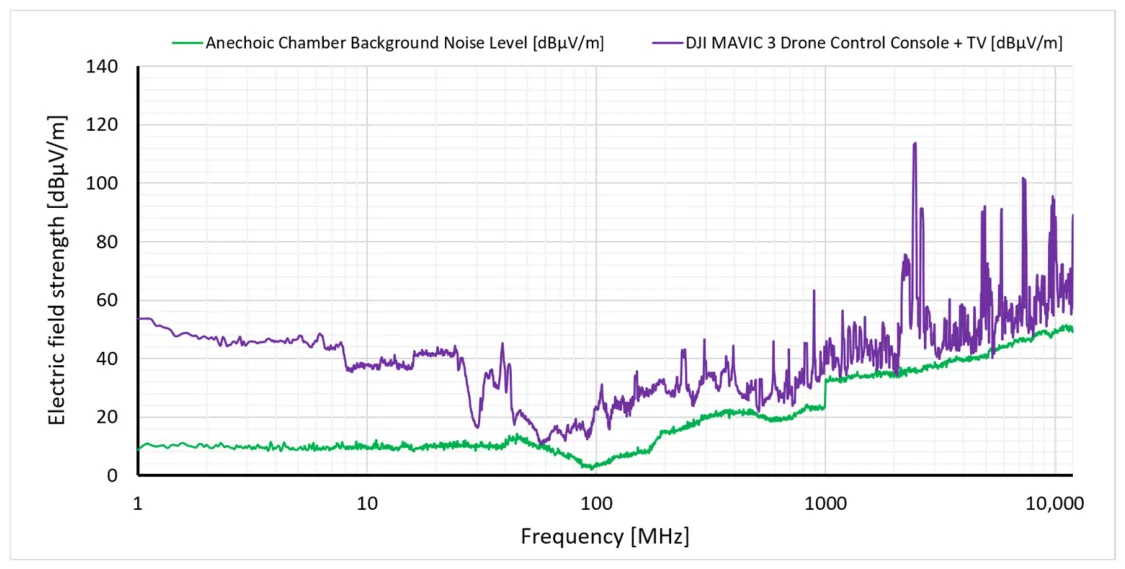
Radiated electromagnetic emission levels generated by the DJI Mavic 3 platform during image transmission to the operator controller and simultaneous image display on an LED television receiver in the frequency range from 1 MHz to 12 GHz.

**Figure 14:**
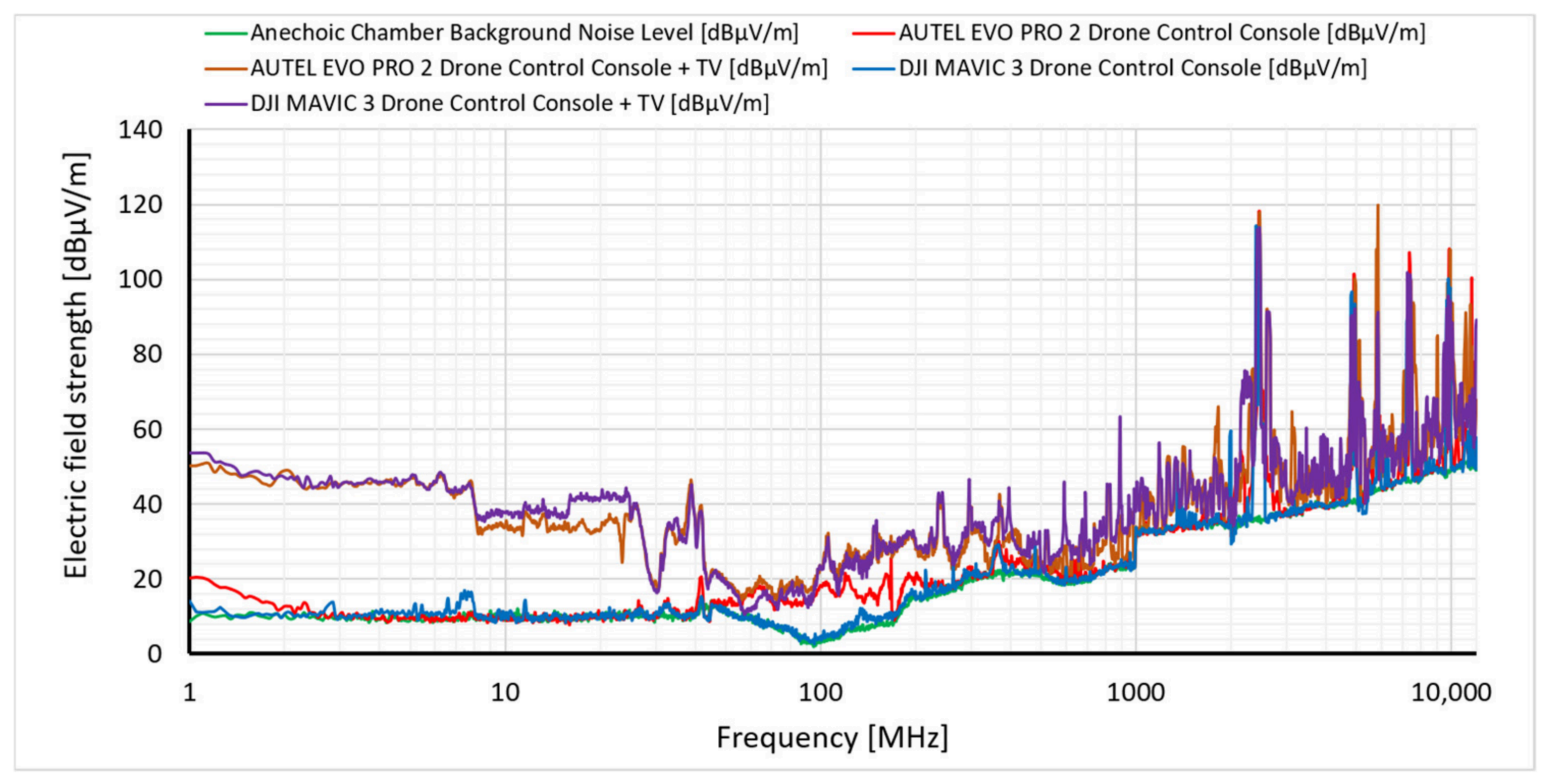
Comparison of radiated electromagnetic emission levels generated by the Autel EVO II Pro and DJI Mavic 3 platforms for the two investigated measurement scenarios over the frequency range of 1 MHz–12 GHz.

**Figure 15:**
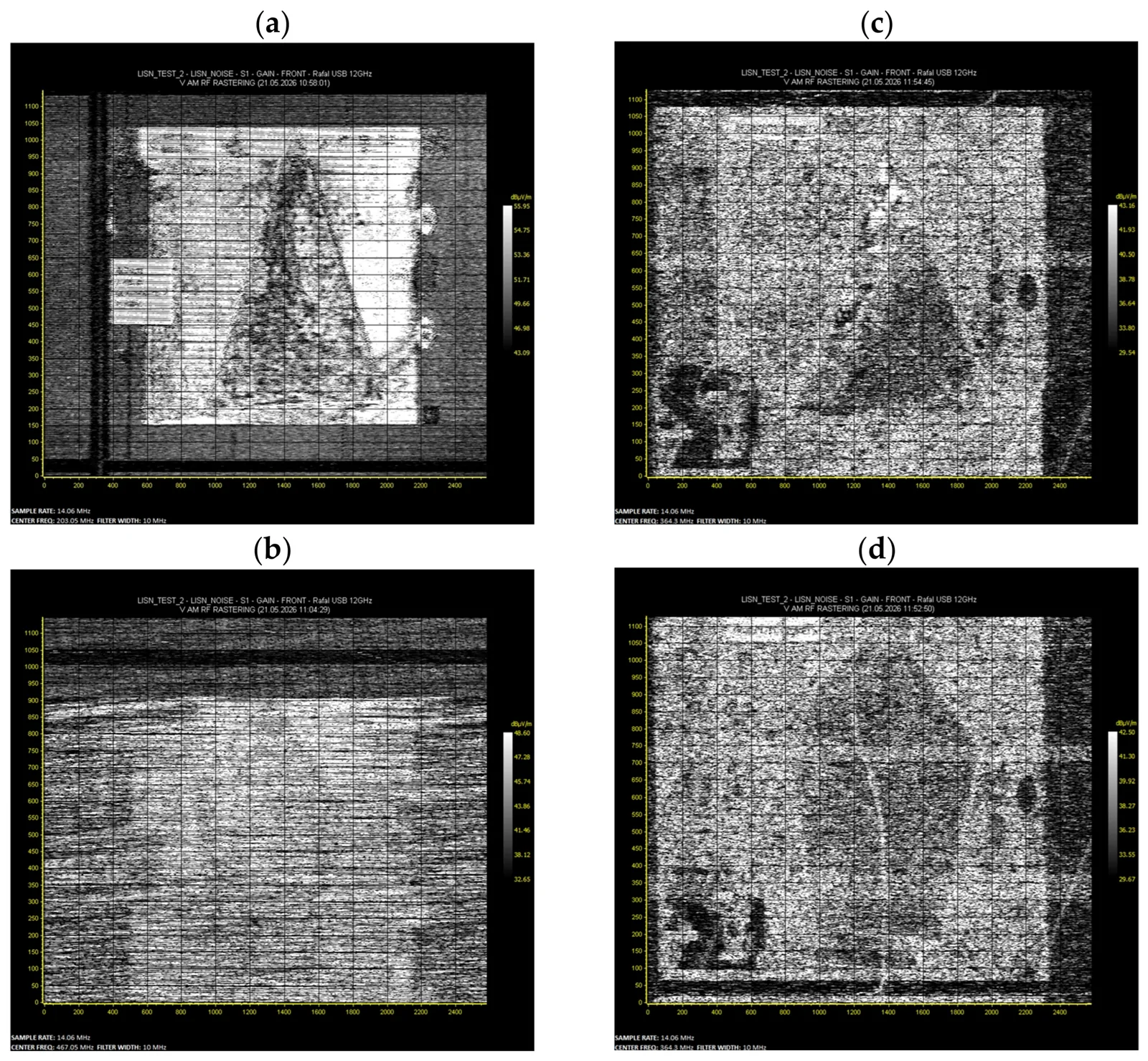
Appearance of reconstructed images transmitted between the flying platform and the controller: (**a**) black triangle on white background—AUTEL EVO II Pro; (**b**) black pentagon on white background—AUTEL EVO II Pro; (**c**) black triangle on white background—DJI Mavic 3; (**d**) black pentagon on white background—DJI Mavic 3.

**Figure 16:**
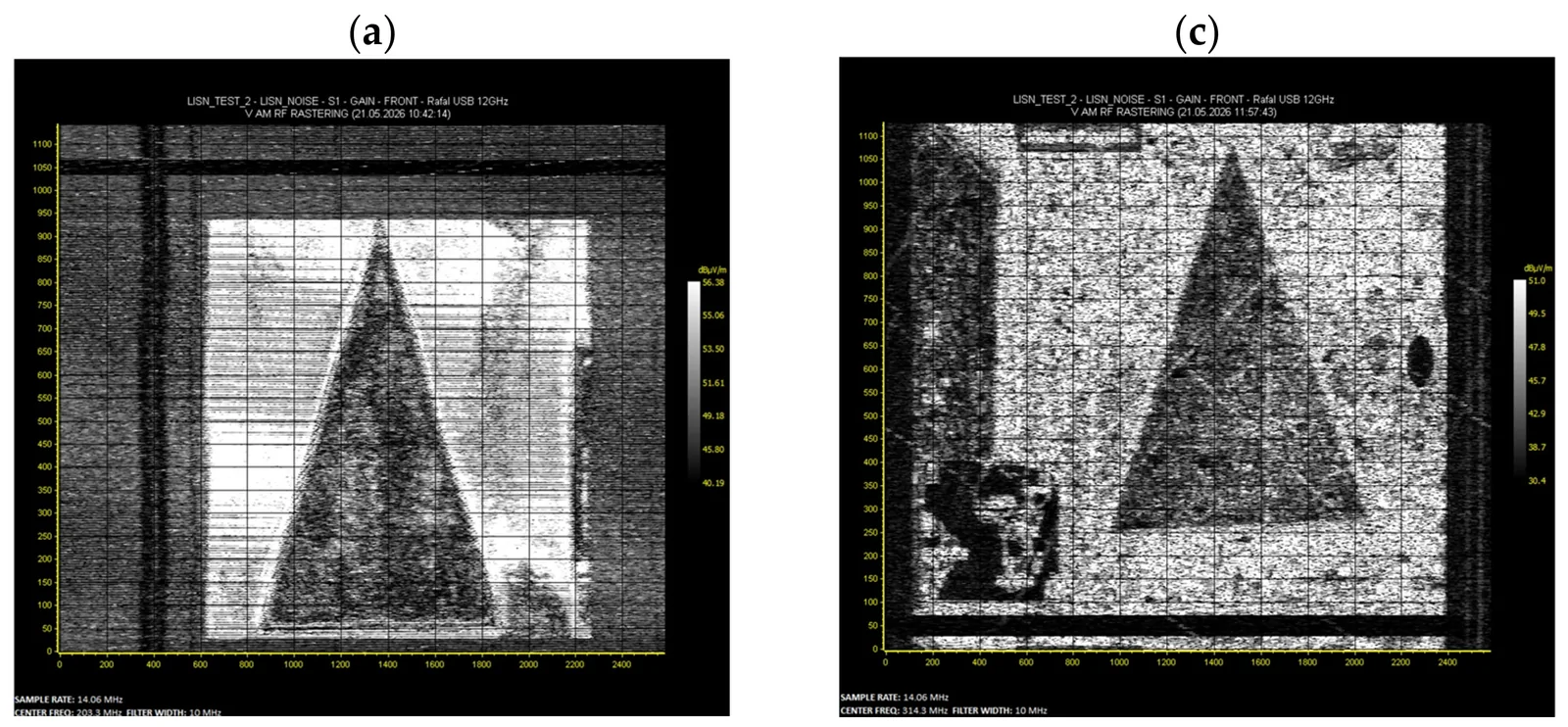

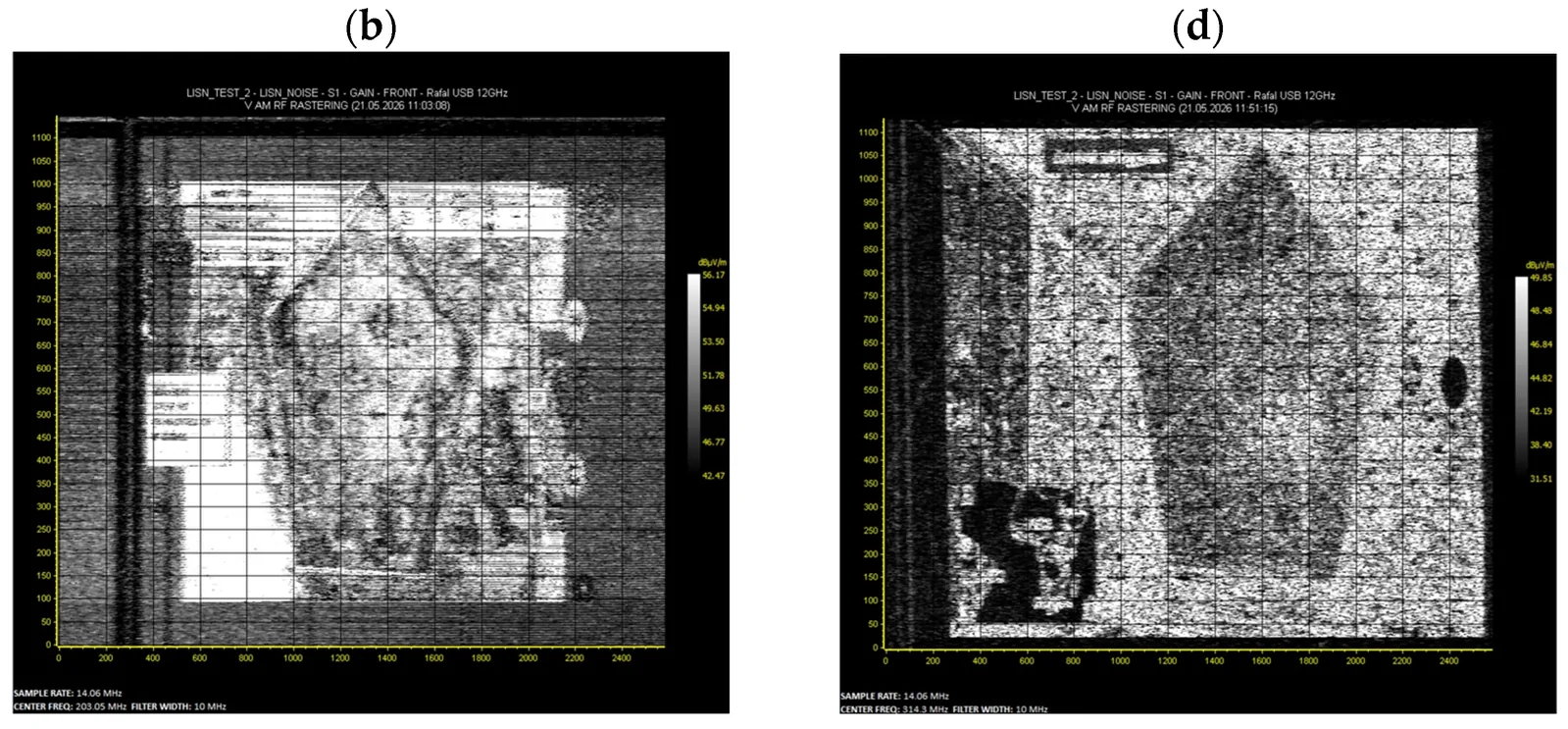
Examples of reconstructed images obtained from recorded electromagnetic emissions during image transmission between the UAV platform and the operator controller connected to an LED television receiver: (**a**) black triangle on a white background—Autel EVO II Pro; (**b**) black pentagon on a white background—Autel EVO II Pro; (**c**) black triangle on a white background—DJI Mavic 3; (**d**) black pentagon on a white background—DJI Mavic 3.

**Figure 17:**
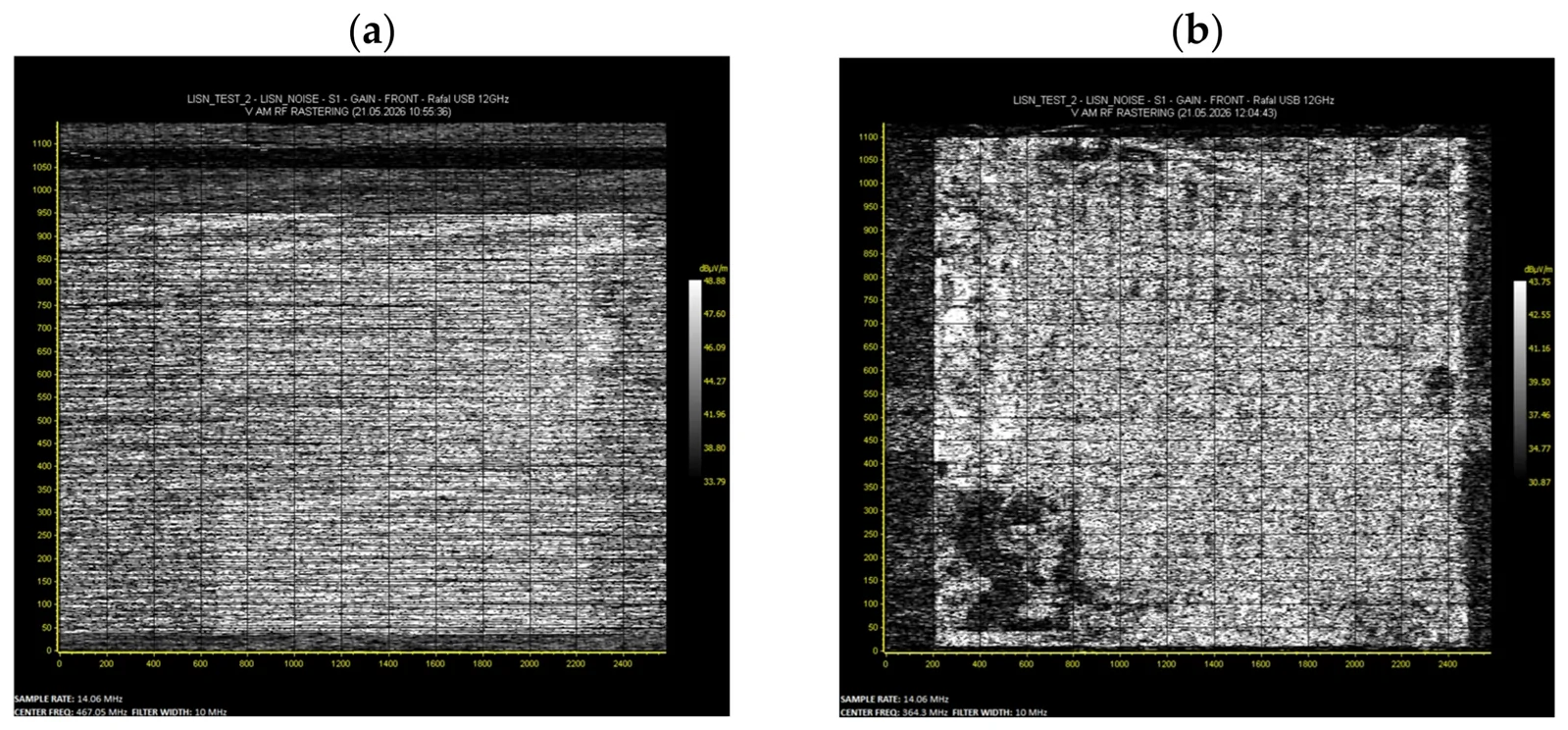
Reconstructed images obtained from electromagnetic emissions during image transmission between the UAV platform and the operator controller: (**a**) high-contrast black-and-white image containing numerical information (“Infiltration Map”)—Autel EVO II Pro, and (**b**) high-contrast black-and-white image containing numerical information (“Infiltration Map”)—DJI Mavic 3.

**Figure 18:**
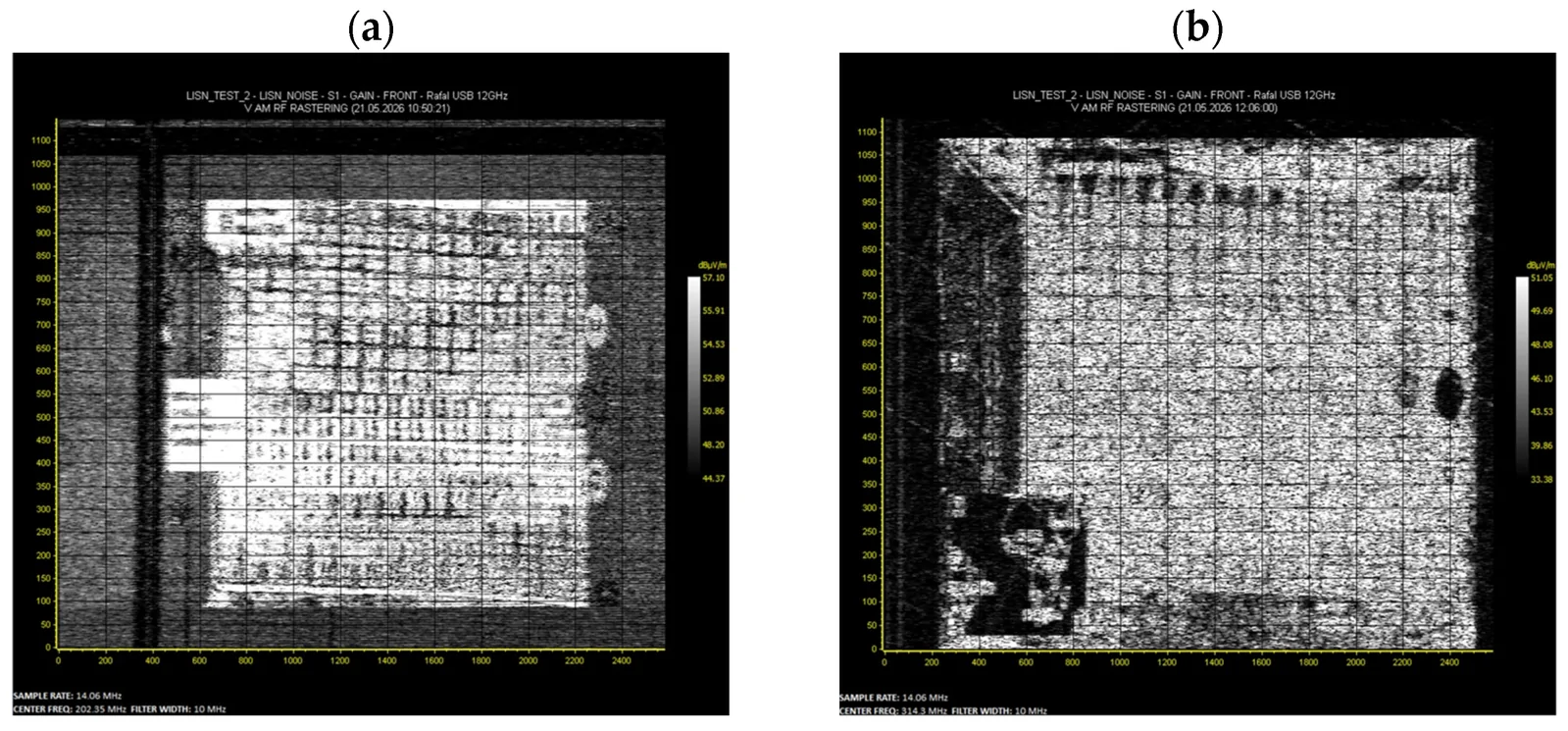
Reconstructed images obtained from electromagnetic emissions during image transmission between the UAV platform and the operator controller connected to an LED television receiver: (**a**) high-contrast black-and-white image containing numerical information (“Infiltration Map”)—Autel EVO II Pro; (**b**) high-contrast black-and-white image containing numerical information (“Infiltration Map”)—DJI Mavic 3.

**Figure 19:**
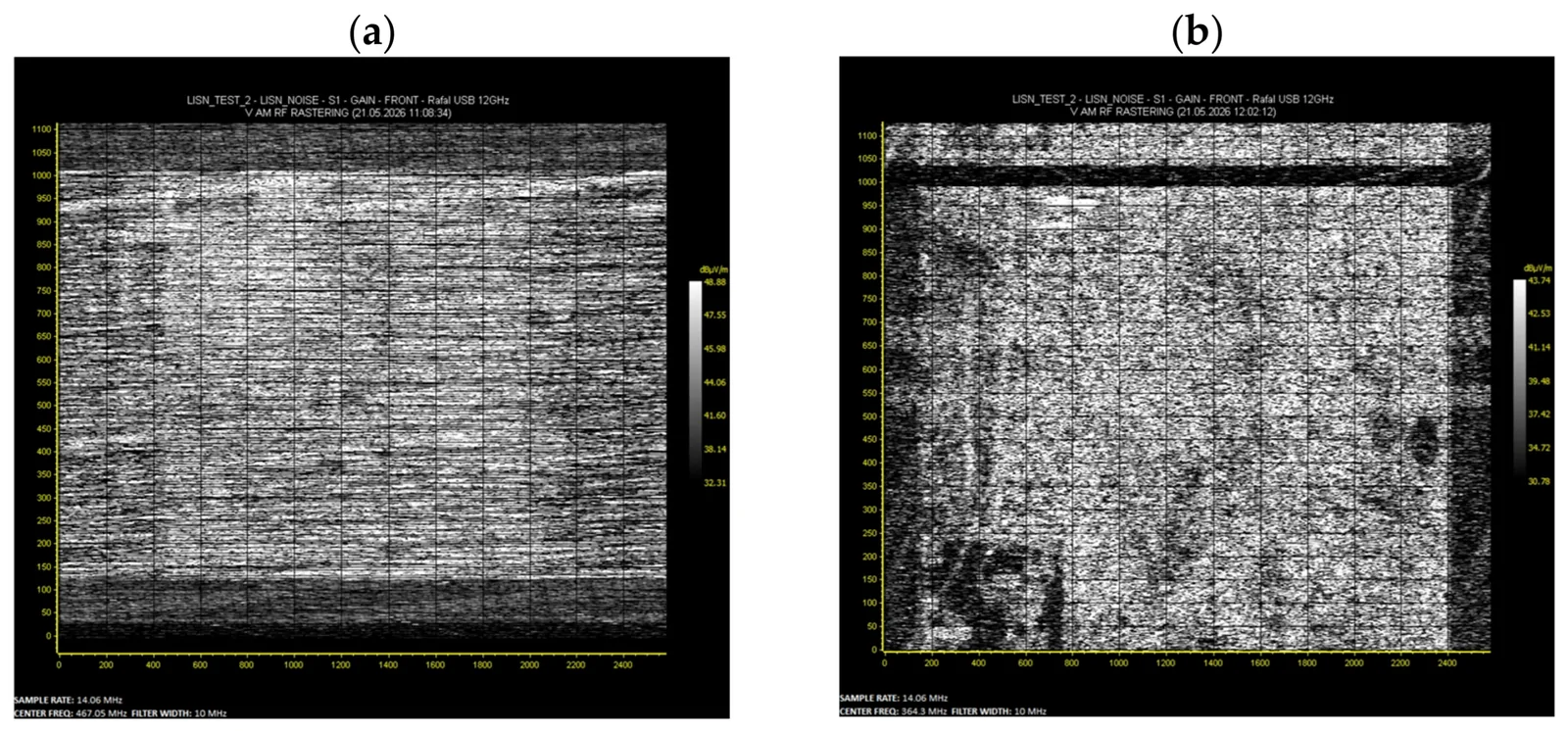
Appearance of reconstructed images transmitted between the flying platform and the controller: (**a**) color 3D robot model—AUTEL EVO II Pro; (**b**) color 3D robot model—DJI Mavic 3.

**Figure 20:**
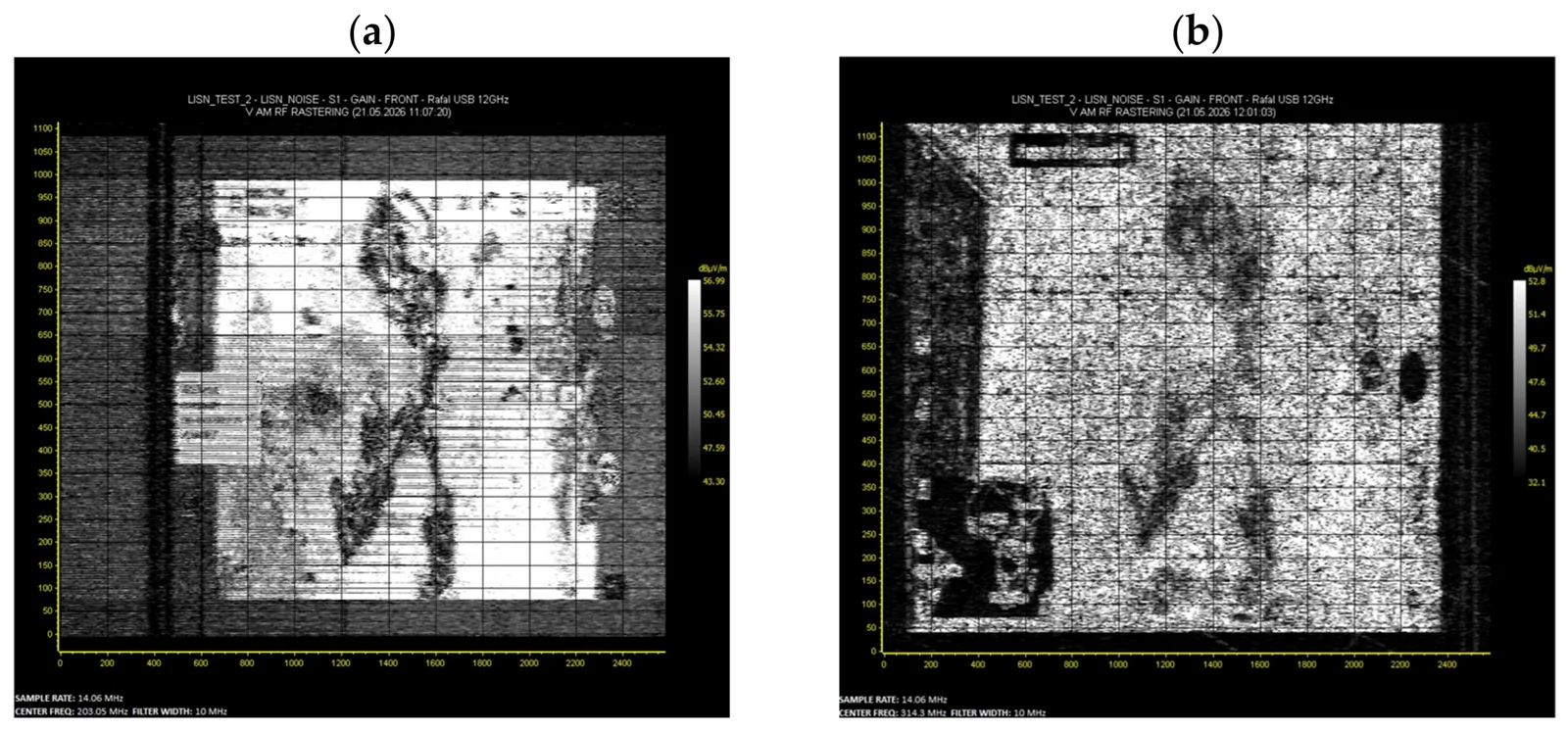
The appearance of the reconstructed images transmitted between the flying platform and the controller connected to the LED TV: (**a**) a colorful 3D robot model—AUTEL EVO II Pro; (**b**) a colorful 3D robot model—DJI Mavic 3.

**Table 1:** Quantitative assessment of the quality of images reconstructed from compromising electromagnetic emanations.

Platform	Image	Configuration	SSIM	PSNR [dB]	Pearson r	IoU	Dice
Autel	Triangle	controller	0.152	8.04	0.539	0.608	0.756
Autel	Triangle	controller + TV	0.196	9.43	0.736	0.844	0.915
Autel	Pentagon	controller	0.050	5.84	0.141	0.445	0.616
Autel	Pentagon	controller + TV	0.137	5.48	0.172	0.422	0.594
Autel	Map	controller	0.025	9.43	0.080	–	–
Autel	Map	controller + TV	0.028	8.61	0.235	–	–
Autel	Robot 3D	controller	0.071	6.85	0.154	–	–
Autel	Robot 3D	controller + TV	0.279	9.25	0.520	–	–
DJI	Triangle	controller	0.061	6.71	0.463	0.560	0.718
DJI	Triangle	controller + TV	0.099	8.60	0.633	0.810	0.895
DJI	Pentagon	controller	0.059	7.17	0.466	0.699	0.823
DJI	Pentagon	controller + TV	0.073	7.48	0.496	0.859	0.924
DJI	Map	controller	0.028	8.85	0.127	–	–
DJI	Map	controller + TV	0.040	8.57	0.159	–	–
DJI	Robot 3D	controller	0.059	6.58	0.219	–	–
DJI	Robot 3D	controller + TV	0.123	8.10	0.425	–	–

## Data Availability

Data available on request due to legal restrictions.

## Abbreviations

The following abbreviations are used in this manuscript:

UAV	Unmanned Aerial Vehicle
EMSEC	Electromagnetic Security
CRT	Cathode Ray Tube
LCD	Liquid Crystal Display
LED	Light-Emitting Diode
CMOS	Complementary Metal-Oxide-Semiconductor
HDMI	High-Definition Multimedia Interface
TMDS	Transition-Minimized Differential Signaling
